# UBR7 inhibits HCC tumorigenesis by targeting Keap1/Nrf2/Bach1/HK2 and glycolysis

**DOI:** 10.1186/s13046-022-02528-6

**Published:** 2022-11-24

**Authors:** Liang Zhao, Min Kang, Xiaomeng Liu, Zhenran Wang, Yan Wang, Haiqiang Chen, Wenhui Liu, Shiqian Liu, Baibei Li, Chong Li, Antao Chang, Bo Tang

**Affiliations:** 1 Department of Hepatobiliary Surgery and Oncology, The First Affiliated Hospital of Guangxi Medical University, No 6 Shuangyong Road, Nanning, 530021 Guangxi People’s Republic of China; 2 Institute of Biophysics, Chinese Academy of Sciences, Beijing, 100101 China; 3 Department of Pancreatic Cancer, Key Laboratory of Cancer Prevention and Therapy, Tianjin Medical University Cancer Institute and Hospital, National Clinical Research Center for Cancer, Tianjin’s Clinical Research Center for Cancer, Tianjin, 300060 China

**Keywords:** Hepatocellular carcinoma, UBR7, Keap1-Nrf2-Bach1 signaling, Glycolysis, Monoubiquitination

## Abstract

**Background:**

Glycolysis metabolism is an attractive target for cancer therapy. Reprogramming metabolic pathways could improve the ability of metabolic inhibitors to suppress cancers with limited treatment options. The ubiquitin–proteasome system facilitates the turnover of most intracellular proteins with E3 ligase conferring the target selection and specificity. Ubiquitin protein ligase E3 component N-recognin 7 (UBR7), among the least studied E3 ligases, recognizes its substrate through a plant homeodomain (PHD) finger. Here, we bring into focus on its suppressive role in glycolysis and HCC tumorigenesis, dependent on its E3 ubiquitin ligase activity toward monoubiquitination of histone H2B at lysine 120 (H2BK120ub).

**Methods:**

In this study, we carried out high-throughput RNAi screening to identify epigenetic candidates in regulating lactic acid and investigated its possible roles in HCC progression.

**Results:**

UBR7 loss promotes HCC tumorigenesis both in vitro and in vivo. UBR7 inhibits glycolysis by indirectly suppressing HK2 expression, a downstream target of Nrf2/Bach1 axis. Mechanically, UBR7 regulates H2BK120ub to bind to *Keap1* promoter through H2BK120ub monoubiquitination, thereby modulating Keap1 expression and downstream Nrf2/Bach1/HK2 signaling. Pharmaceutical and genetic inhibition of glycolytic enzymes attenuate the promoting effect of UBR7 deficiency on tumor growth. In addition, methyltransferase ALKBH5, downregulated in HCC, regulated UBR7 expression in an m6A-dependent manner.

**Conclusions:**

These results collectively establish UBR7 as a critical negative regulator of aerobic glycolysis and HCC tumorigenesis through regulation of the Keap1/Nrf2/Bach1/HK2 axis, providing a potential clinical and therapeutic target for the HCC treatment.

**Supplementary Information:**

The online version contains supplementary material available at 10.1186/s13046-022-02528-6.

## Background

According to the latest global cancer statistics (2020), liver cancer has become the seventh most commonly diagnosed cancer and the third leading cause of cancer death worldwide. Hepatocellular carcinoma (HCC) comprises 75%-85% of the cases [1]. The heterogeneity of the disease poses immense challenges in developing effective diagnostic methods and treatments. In this case, it is an urgent matter to clarify the molecular mechanisms underlying the oncogenesis and malignant features of HCC.

Ubiquitination is a highly conserved and tightly controlled post-translational modification in eukaryotes. As critical players in the ubiquitination process, E3 ubiquitin ligases selectively transfer the ubiquitin from the E2 enzyme to substrate and confer specificity to ubiquitination [2–4]. The ubiquitin protein ligase E3 component N-recognin (UBR) family is a unique class of E3 ubiquitin ligases characterized by a 70-residue zinc finger-type UBR-box domain, which is essential for recognition of N-degrons, the N-terminal residues that destabilize a protein [5, 6]. So far, seven UBR box E3 ligases have been identified in mammals. Despite the presence of a UBR-box, UBR7 does not recognize the canonical N-degron sequence [7]. Instead, it has evolved with an atypical plant homeodomain (PHD) finger harboring a unique Cys4-His2-Cys2 motif as a putative chromatin-binding module that does not exist in any other UBR family members [8, 9]. Recently, UBR7 has been found to monoubiquitinate histone H2B or act as a histone chaperone for post-nucleosomal histone H3[ 9–11]. Moreover, a novel nucleotide biosynthesis regulatory role for UBR7 has been identified in NOTCH1-driven T cell acute lymphoblastic leukemia [12, 13]. Besides these findings, the cellular functions of UBR7 and its role in tumorigenesis remain largely uncharacterized.

Aerobic glycolysis or the Warburg effect was originally found in liver cancer [14], characterized by enhanced glucose uptake and lactate production, which is implicated in the regulation of angiogenesis, drug resistance, immune evasion, metastasis and proliferation in HCC [15]. Hexokinase (HK) is one of the three rate-limiting enzymes in aerobic glycolysis and catalyze the first important irreversible step by phosphorylating glucose. HK2 is the most efficient isoform at promoting this process with well-established regulatory mechanisms [16]. HK2 is highly expressed in HCC tissues and has been directly linked to clinical stages and poor clinical outcomes. HK2 silencing enhances the sensitivity of HCC to drugs like metformin or sorafenib [17–19]. Thus, HK2 is considered to be a highly promising metabolic target for HCC treatment.

In the present study, we demonstrate that UBR7 exerts the tumor suppressive function in HCC through targeting Keap1/Nrf2/Bach1/HK2 axis and aerobic glycolysis, which depends on its E3 ubiquitin ligase activity toward monoubiquitination of histone H2B at lysine120 (H2BK120Ub).

## Methods

### Mice

The UBR7^fl/fl^ mice were purchased from Cyagen Biosciences Inc. (Guangzhou, China). The liver tissue-specific UBR7 knockout mice were obtained by crossing UBR7^fl/fl^ mice with Alb-Cre mice. One year later, Alb-Cre;UBR7^fl/fl^ mice were dissected, and the tumour in the liver was observed and stained with H&E. In addition, the survival time of Alb-Cre;UBR7^fl/fl^ mice was analysed. 8-week-old mice (WT and Alb-Cre;UBR7^fl/fl^) were used to extract primary liver cells. All mice have a C57BL/6 genetic background. All mice were maintained in SPF conditions. All mice used in this study are male. All animal experiments were conducted in accordance with the care and guidelines for laboratory animals of the National Institutes of Health.

### Cell lines

The human HCC cell line Huh-7 was from Cell Resource Center, Shanghai Institutes for Biological Sciences, Chinese Academy of Sciences, MHCC-97L was from the Liver Cancer Research Institute of Zhongshan Hospital of Fudan University (Shanghai, China), BEL-7402 and BEL-7404 were purchased from Shanghai Aolu Biotechnology Co., Ltd. (Shanghai, China). HepG2 cell line was purchased from ATCC (American Type Culture Collection, ATCC). All hepatocellular carcinoma cells were cultured with DMEM (Biological Industries, Israel) and 10% FBS (Biological Industries, Israel) medium. The cell culture environment was 37 °C with 5% CO_2_ and saturated humidity.

### High-throughput screening

In the high-throughput screening, a lentiviral library (3D-HTS technology) of 1122 unique shRNAs targeting 561 human epigenetic modifiers was used. In the initial screening, 1 × 10^3^ MHCC-97L cells were seeded in each well of a 96-well plate and infected with different lentiviruses in an arrayed manner. Seventy-two hours after infection, cells were treated with 3% O_2_. The supernatant was collected and the concentration of secreted lactic acid was determined by ELISA. In the second screening, three separate siRNAs were designed and synthesized for the candidate genes, transfected into MHCC-97L cells, and treated as in the initial screening. A gene is considered positive if it is scored by at least two siRNAs.

### RNA-seq analysis

Eight-week-old WT and Alb-Cre;UBR7^fl/fl^ mice were used, first dissecting the mouse liver tissue, then cutting the liver tissue into small pieces, and finally digesting and extracting primary liver cells. The TRIzol RNA reagent was then used to extract total RNA from primary liver cells. cDNA library construction and sequencing were performed on the Illumina HiSeq platform. Bowtie2 was used to compare high-quality readings with the human reference genome (GRCh38). Differential genes were analysed by DESeq2 software.

### Lactate Assay, Glucose Uptake Assay and ATP Assay

Lactate assay kit (ab65330), Glucose Uptake Assay (ab136955) and ATP assay (ab83355) were purchased from Abcam. The detection of lactate, glucose uptake and ATP levels were carried out according to the method recommended by the kit.

### In vivo tumorigenesis

HepG2 cells overexpressing or knocking out UBR7 were injected subcutaneously into nude mice. The volume of xenograft tumours was measured weekly. After 32 days, the xenograft tumour was taken out and weighed.

### MTT and Colony formation

The appropriate amount of HCC cells was seeded in 96-well plates. 0.5 mg/ml MTT was added when the cells were cultured to 12 h, 24 h, 48 h and 72 h. After incubating for 4 h, 150 μl of DMSO was added and mixed. Measure the OD value of each period using a spectrophotometer. An appropriate amount of HCC cells was seeded in 6-well plates, cultured for 14 days, and the medium was changed twice a week. The cell clone forming ability was photographed through a microscope and the number of clones larger than 25 μm was counted.

### Methylated RNA immunoprecipitation qPCR (MeRIP-qPCR)

The MeRIP-qPCR assay was performed to determine the m^6^A level of UBR7. Total intracellular RNA was extracted by using TRIzol reagent. Anti-m^6^A antibodies or anti-immunoglobulin G (IgG; Cell Signaling Technology) (3 µg) was first conjugated to protein A/G magnetic beads and mixed with 100 µg aliquot of total RNA in IP buffer containing RNase/protease inhibitors. m^6^A-modified RNA was eluted twice with 6.7 mM N^6^-methyladenosine 5’-monophosphate sodium salt at 4 ºC for 1 h.

### Statistical analysis

Statistical analyses were performed using SPSS software. Statistical significance between the means of a minimum of three groups was determined using unpaired two-tailed Student’s t test, two-way ANOVA, or linear regression analysis. The data are expressed as an average ± S.D. *p* < 0.05 was considered statistically significant. All results are representative of at least three independent experiments.

## Results

### RNAi screening identifies epigenetic candidates in regulating lactic acid

**Fig. 1:**
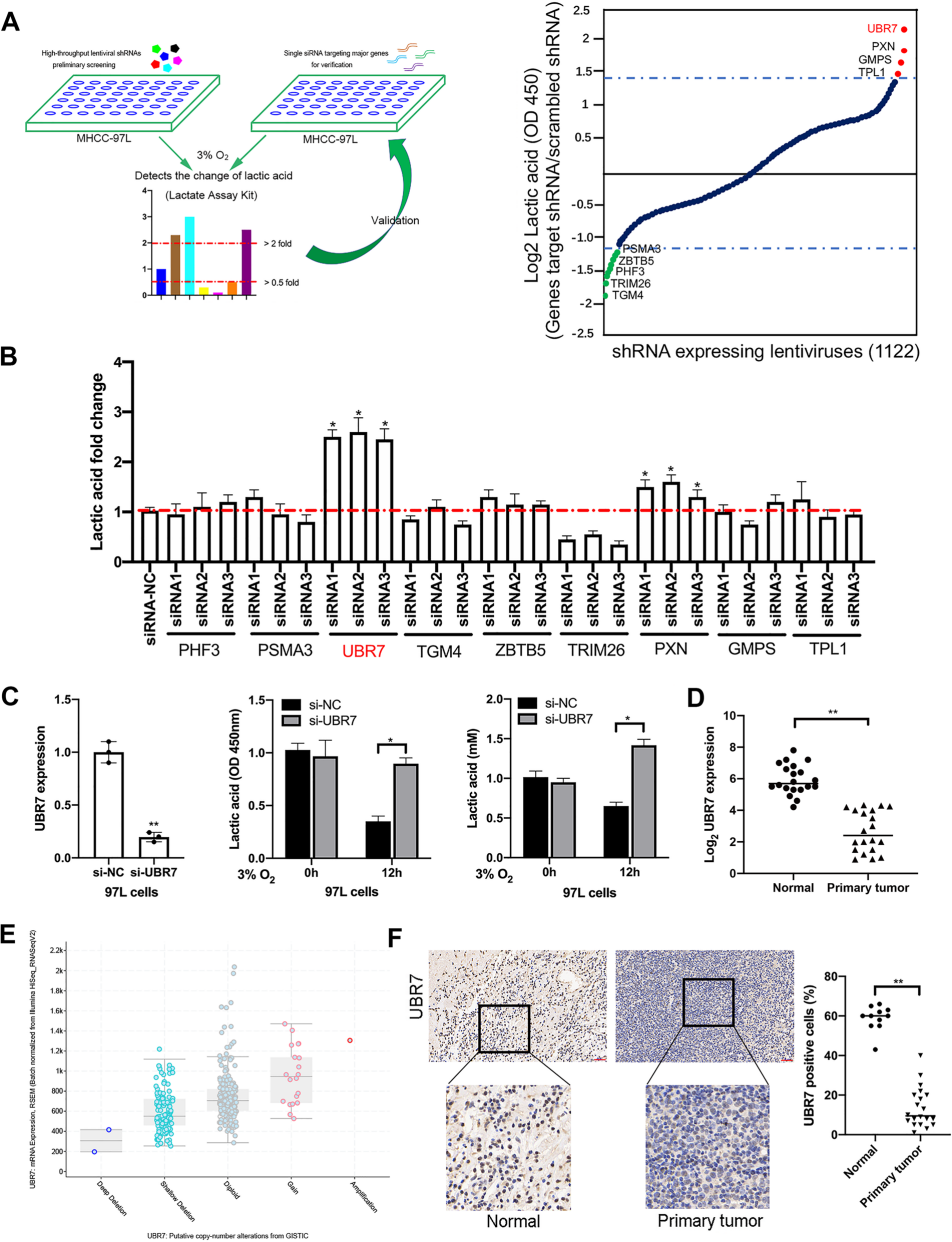
RNAi screening identifies epigenetic candidates regulating the change of lactic acid. **A** Left: a two-stage screening strategy diagram of a lentivirus-mediated shRNA library and siRNA. Right: the fold change of lactate in MHCC-97L cells transfected with 1,122 targeted shRNA followed by 3% O_2_ treatment for 12 h. Targeting genes that significantly increase or decrease lactate changes are highlighted in red or green, respectively. **B** Fold changes of the lactate of MHCC-97L cells transfected with siRNAs for secondary validation followed by 3% O_2_ treatment as in (**A** Right). **C** qRT-PCR analysis of UBR7 expression (left), and ELISA of Lactic acid secretion (right, middle) of 97L cells transfected with scrambled siRNA (si-NC) or siRNA specific for UBR7 (si-UBR7), followed by 3% O_2_ treatments as in (**A** Right). **D** qRT-PCR analysis of UBR7 expression in HCC tissues (*n* = 20) and normal liver tissues (*n* = 20). **E** The UBR7 gene copy-number is analysed in the tissues of patients with hepatocellular carcinoma in the GISTIC database. **F** IHC staining and statistics of UBR7 protein expression levels in liver cancer tissues and normal liver tissues. Data are shown as mean ± SD of three independent experiments. *p < 0.05, **p < 0.01. The scale bar in **F** is 100 μm

To explore the epigenetic regulators of glycolysis in HCC cells, we performed a cell-based high-throughput small hairpin RNA (shRNA) screening in MHCC-97L cells by using lactate assay kit to measure the change of lactate acid in the supernatant after 3% O_2_ treatment for 12 h. The screening library consists of 1,122 unique shRNA-expressing lentiviruses, targeting 561 candidate genes encoding modulators of chromatin structure and function (Fig. 1A). According to the fold changes in lactate acid secretion after shRNA transfection, 9 genes were identified in the primary screening, including UBR7, silencing of which enhanced lactate acid change by more than twofold. Meanwhile, knockdown of the other candidate genes, including PXN, GMPS, TPL1 also led to elevated lactate acid levels, whereas silencing of the other 5 genes, including PSMA3, ZBTB5, PHF3, TRIM26, and TGM4, inhibited lactate acid generation. A secondary screen was then conducted to further validate the 9 primary hits by single small interfering RNAs (siRNAs) targeting each candidate. Three individual siRNAs were specifically designed for each gene, and a positive hit was defined as significant changes in lactate acid secretion caused by transfection of at least two siRNAs. After the secondary screen, only UBR7 and PXN came out as positive hits (Fig. 1B).

Subsequently, our preliminary functional screening showed that lactate acid secretion was significantly higher in UBR7-silenced MHCC-97L cells compared to that in control cells in response to 3% O_2_ treatment for 12 h (Fig. 1C). Similar elevation of the lactate acid secretion was also observed in the UBR7-silenced Huh-7 and HepG2 cells transiently transfected by siRNA (Supplementary Fig. 1B-D). Previous studies have reported the importance of zinc-coordinating His163 and His166 in the catalytic activity of UBR7, the mutation of which would affect the E3 ubiquitin ligase activity toward monoubiquitination of histone H2B [9]. To further confirm the role of UBR7 in HCC glycolysis, BEL-7402 cells expressing wild-type (UBR7-WT) and H163S/H166S catalytic-mutant (UBR7-CM) UBR7 were constructed and functional screening was performed. Obviously, overexpression of UBR7-WT, but not UBR7-CM, significantly inhibited the lactate acid secretion in BEL-7402 cells, suggesting that the inhibitory effect of UBR7 on lactate acid levels was probably dependent on its E3 ubiquitin ligase activity toward monoubiquitination of histone H2B **(**Supplementary Fig. 1A).

Furthermore, by comparing the mRNA level of UBR7 in HCC tissues (*n* = 20) and normal liver tissues (*n* = 20), we found that the expression of UBR7 was significantly lower in primary tumors than that in normal tissues (Fig. 1D), which is consistent with the putative lower gene copy-number of UBR7 in tumor samples from GISTIC database (Fig. 1E). In contrast to normal adjacent tissues, UBR7 protein levels were notably lower in primary tumor tissues, implying the potential tumor-suppressive activity of UBR7 in HCC (Fig. 1F). Taken together, these data suggest that UBR7 plays a potent role in regulation of lactate acid levels in HCC, which might contribute to tumor suppression.

### UBR7 inhibits HCC proliferation and tumorigenesis

**Fig. 2:**
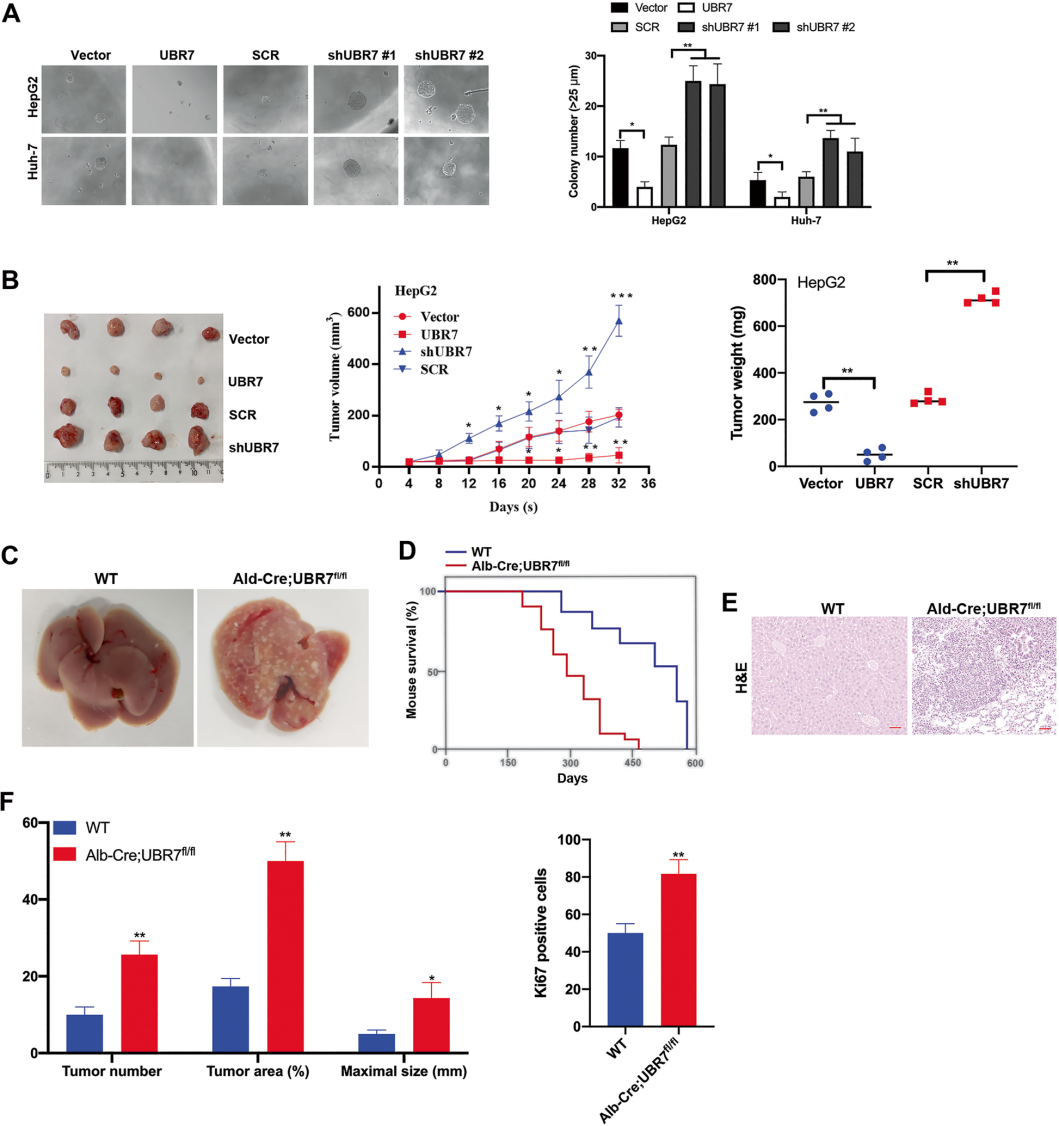
UBR7 inhibits HCC proliferation and tumorigenesis. **A** The clone formation sphere experiment analysed Huh-7 and HepG2 cells overexpressing or knocking out UBR7. **B** The overexpression or knockout of UBR7 cells constructs xenogeneic tumours subcutaneously in nude mice (left). Tumour volumes (mm^3^) (middle) and the weight (right) of mouse xenografts implanted with HepG2 cells. **C** The liver morphology of UBR7^WT^ (WT) and Alb-Cre;UBR7^fl/fl^ mice. **D** Survival analysis of UBR7^WT^ (*n* = 24) and Alb-Cre;UBR7^fl/fl^ mice (*n* = 19). **E** Representative H&E staining images for sections of livers in UBR7^WT^ and Alb-Cre;UBR7^fl/fl^ mice. **F** Quantification of tumour area (%), number, maximal size (mm) (left), and Ki67 positive cells (%) (right) in UBR7^WT^ and Alb-Cre;UBR7^fl/fl^ mice. Data are shown as mean ± SD of three independent experiments. *p < 0.05, **p < 0.01, ***p < 0.01. The scale bar in **A **and **E** was 100 μm

To characterize the influence of UBR7 on cell proliferation, we compared UBR7 silencing and overexpression in HCC cell lines. HepG2 and Huh-7 cells were transfected with lentiviral vectors encoding human UBR7 inserts or shRNAs, and the expression of UBR7 was measured by immunoblotting (Supplementary Fig. 2A, 3B). UBR7 overexpression apparently inhibited the viability and colony formation of HCC cells, as determined by MTT and colony formation assays; whereas deficiency of UBR7 led to an increase of viability and colony formation in HCC cells (Fig. 2A**,** Supplementary Fig. 2C-F). Importantly, such inhibitions caused by UBR7 overexpression were not obvious in HCC cells transfected with UBR7-CM, which again highlighted the importance of UBR7’s catalytic activity on its tumor-suppressive function (Supplementary Fig. 2G, 2H). UBR7 overexpression significantly inhibited cell invasion while UBR7 knockdown increased cell invasion in HepG2 and Huh7 cells (Supplementary Fig. 2I, 2 J). Moreover, UBR7 overexpression promoted cell apoptosis, but UBR7 silencing had the vice versa effect (Supplementary Fig. 2 K). Consistent with the above in vitro observations, xenograft mice implanted with HepG2 cells bearing UBR7 inserts exhibited reduced tumor volume and weights compared to control models, whereas exceeding tumor formation was found in xenografts established with injection of UBR7-deficient HepG2 cells (Fig. 2B). Taken together, these data implied that UBR7 might suppress HCC tumorigenesis by inhibiting cell viability, colony formation, proliferation and cell invasion.

To further explore the function of UBR7 on HCC tumorigenesis, we generated liver-specific UBR7 knockout mice (Alb-Cre; UBR7^fl/fl^ mice) by crossing UBR7^fl/fl^ mice with albumin-Cre mice, and UBR7 silencing in the liver was confirmed by immunoblotting and IHC staining (Supplementary Fig. 3A, 3B). At 9 months of age, Alb-Cre; UBR7^fl/fl^ mice developed visible tumors, which were barely seen in their WT littermates (Fig. 2C). Compared with their control littermates, Alb-Cre; UBR7^fl/fl^ mice displayed notably shorter survival (Fig. 2D). Histologically, H&E staining of livers from 9-mo-old Alb-Cre; UBR7fl/fl mice showed apparent HCC features like clear-cell dysplasia. (Fig. 2E). Additionally, Alb-Cre; UBR7^fl/fl^ mice exhibited faster disease progression with increased tumor numbers, tumor areas, maximal sizes and Ki67 expression (Fig. 2F). Collectively, the above findings indicate that UBR7 depletion promotes HCC tumorigenesis both in vitro and in vivo.

### UBR7 inhibits glycolysis by negatively regulating HK2

**Fig. 3:**
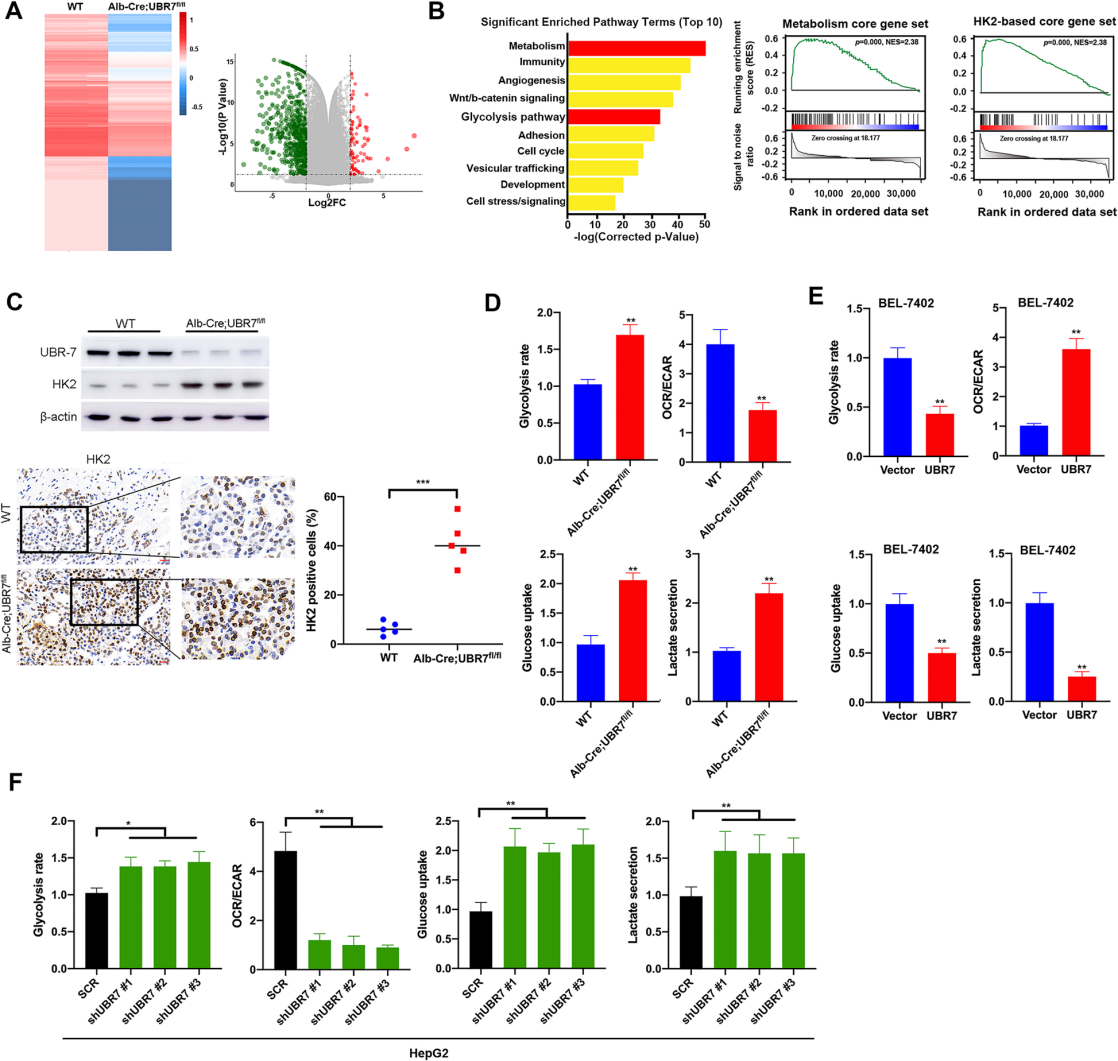
UBR7 inhibits glycolysis by negatively regulating HK2. **A** Left: heatmap summarising genes differentially expressed in the Alb-Cre;UBR7^fl/fl^ livers compared to UBR7^WT^ (WT) livers via RNA-sequencing (RNA-seq) analysis. Right: volcano plots of the differentially expressed genes with 104 up-regulated genes and 369 down-regulated genes. **B** Left: Gene Ontology (GO) analysis showed different genes in (**A**). Right: Gene Set Enrichment Analysis (GSEA) output images of two chosen pathways displaying a correlation of differentially regulated genes in Alb-Cre;UBR7^fl/fl^ liver with the “Metabolism core gene” set and “HK2-based core gene” set. **C** Immunohistochemical and Western blot analysis of the expression level of HK2 in Alb-Cre;UBR7^fl/fl^ and WT liver. **D** WT and Alb-Cre;UBR7^fl/fl^ mouse primary liver cells were analysed for glycolysis efficiency, the oxygen consumption rate (OCR), glucose uptake and lactate secretion levels. **E**, **F** Glycolysis efficiency, the oxygen consumption rate (OCR), glucose uptake and lactate secretion levels were detected in UBR7 overexpressing BEL-7402 or UBR7 knockout HepG2 cells. Data are shown as mean ± SD of three independent experiments. *p < 0.05, **p < 0.01, ***p < 0.001. The scale bar in (**C**) is 100 μm

To gain insight into potential mechanisms contributing to the UBR7-suppressed liver tumorigenesis, RNA-sequencing (RNA-seq) transcriptome analyses were performed with hepatocytes isolated from WT and Alb-Cre; UBR7^fl/fl^ mice. As a result, a total of 104 genes were significantly up-regulated, and 369 genes were down-regulated in liver of Alb-Cre; UBR7^fl/fl^ mice (Fig. 3A). As shown in Fig. 3B, among the top 10 enriched Gene Ontology (GO) pathways, both metabolism and glycolysis pathways stood out as one of the most significantly enriched ones, suggesting the essential role of UBR7 in mediating metabolism and glycolysis. Besides the metabolism core gene set regulated by UBR7-dependent transcription in HCC, Gene Set Enrichment Analysis (GSEA) also uncovered the HK2-based signature under regulation of UBR7. Given that HK2 is highly expressed in HCC and is the most efficient isoform of hexokinase in promoting aerobic glycolysis, we next investigate whether UBR7 could mediate the HK2 expression in HCC. Apparently, increased expression of HK2 was observed in liver cells of Alb-Cre; UBR7^fl/fl^ mice compared to their WT littermates **(**Fig. 3C). Similarly, in HepG2 and Huh-7 cells, UBR7 silencing elicited upregulation of HK2, whereas its overexpression caused downregulation of HK2 (Supplementary Fig. 4A-D). However, Co-IP analysis revealed no notable difference in ubiquitination of HK2 between scramble and UBR7-overexpressing cells, supporting that HK2 might not be a direct target of UBR7 (Supplementary Fig. 4E).

Since both metabolism and glycolysis were identified as the most significantly altered pathways in UBR7 knock-out hepatocytes, we next examined whether UBR7 was a crucial factor in regulating HCC metabolism, especially glycolysis. Primary liver cells from WT and Alb-Cre; UBR7^fl/fl^ mice were thus analyzed for glycolysis efficiency, in terms of the oxygen consumption rate (OCR), glucose uptake and lactate secretion levels. As expected, elevated glycolysis rate and glucose uptake were found in UBR7^fl/fl^ mouse hepatocytes. Moreover, UBR7^fl/fl^ mouse hepatocytes exhibited significantly reduced OCR and lactate secretion (Fig. 3D). Similar results were found in UBR7-deficient HepG2 and BEL-7404 cells (Fig. 3F, Supplementary Fig. 4G), whereas the opposite trend was observed upon excessive expression of UBR7 in BEL-7402 and BEL-7404 cells (Fig. 3E, Supplementary Fig. 4F). These findings altogether imply that UBR7 might inhibit glycolysis by indirectly suppressing HK2 expression in HCC.

### HK2-stimulated glycolysis mediates the UBR7 deficiency-induced metastasis and invasive phenotype

**Fig.4:**
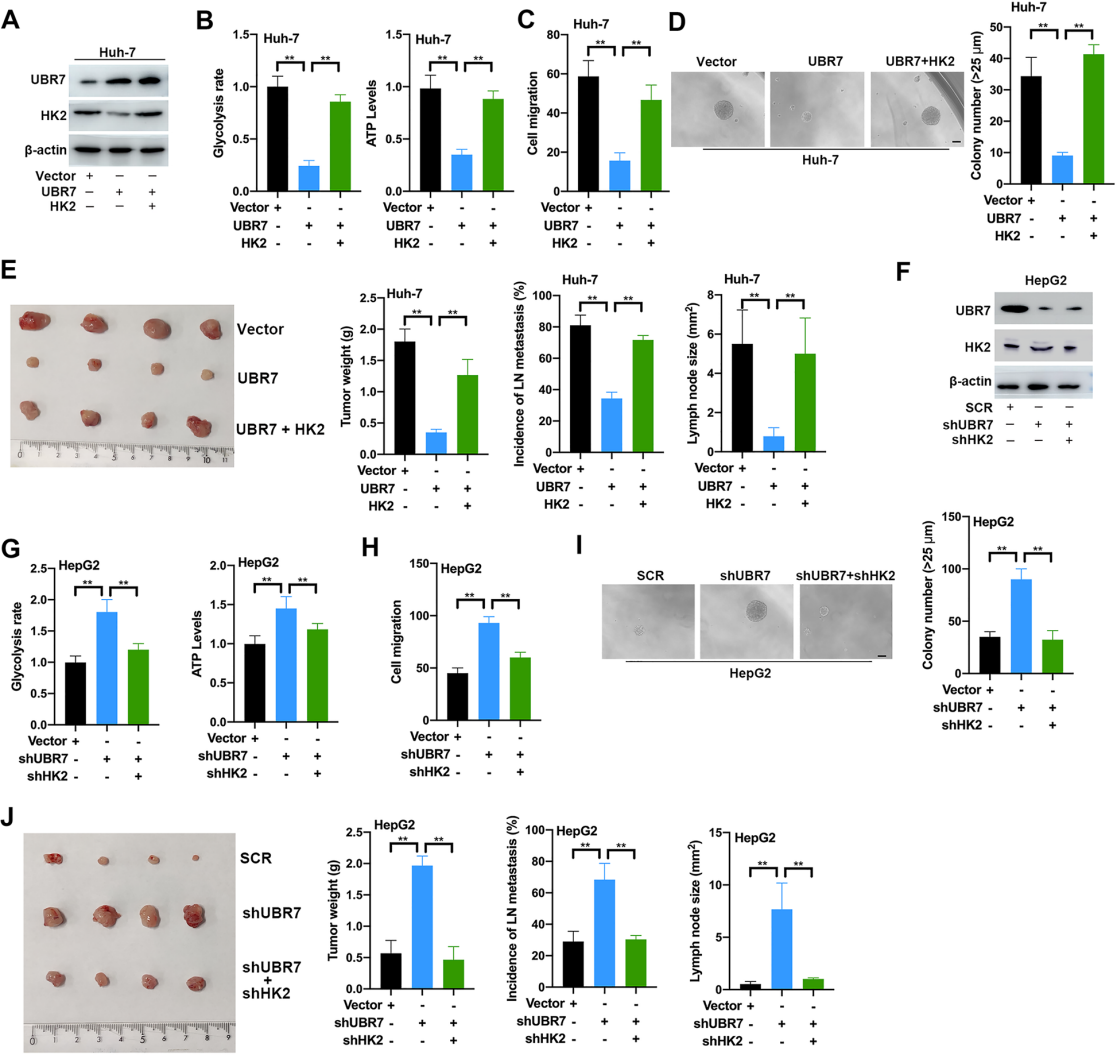
HK2-stimulated glycolysis mediates the UBR7 deficiency-induced metastatic and invasive phenotype. **A** Western blot detected Huh-7 cells stably overexpress UBR7 and HK2. **B-D** Glycolysis rate, ATP level, cell migration and clone formation ability were detected in Huh-7 cells overexpressing UBR7 or/and HK2. (**E**) Left: Xenograft tumour and tumour weight were constructed by the subcutaneous injection of Huh-7 cells overexpressing UBR7 and HK2 alone or together. Right: incidence of LN metastasis and Lymph node size level after Huh-7 cell tail vein injection. (**F**)Western blot detected HepG2 cells knockout UBR7 or/and HK2. **G-I** Glycolysis rate, ATP level, cell migration and clone formation ability were detected in HepG2 cells with the knockout of UBR7 or/and HK2. **J** Left: Xenograft tumour and tumour weight were constructed by subcutaneous injection of HepG2 cells knocking-out UBR7 and HK2 alone or together. Right: incidence of LN metastasis and Lymph node size level after HepG2 cells knocking-out UBR7 and HK2 alone or together in the tail vein injection. Data are shown as mean ± SD of three independent experiments. *p < 0.05, **p < 0.01. The scale bar in (**D**) and (**I**) was 100 μm

The inhibitory effects of UBR7 on HK2 expression, aerobic glycolysis and tumorigenesis in hepatocytes prompted us to further explore the underlying connection. As UBR7 negatively regulate HK2, a critical rate-limiting enzyme in aerobic glycolysis, we thus set out to examine whether UBR7 exerts its function depending on HK2 in HCC. To this end, stable Huh-7 cell lines expressing both UBR7 and HK2 inserts were established (Fig. 4A). Obviously, forced expression of HK2 almost completely rescued the glycolysis rate and ATP levels in Huh-7 cells with excessive expression of UBR7 (Fig. 4B). Meanwhile, cell migration and colony formation abilities of Huh-7 cells were also significantly reversed by HK2 overexpression in UBR7 background (Fig. 4C,D). Similar results were obtained in BEL-7402 cells transfected with the same inserts (Supplementary Fig. 5). As shown in (Fig. 4E**)**, the tumor size, tumor weight, incidence of lymph node metastasis, and lymph node size were evaluated in the mouse xenografts injected with the double transfected Huh-7 cells. The in vivo evidence also revealed that HK2 overexpression abolished the growth-inhibitory effect elicited by UBR7 overexpression.

Likewise, stable HepG2 cell lines transfected with both UBR7 and HK2 shRNAs were also established to test the role of UBR7-HK2 axis in glycolysis, as well as cell proliferation and carcinogenesis (Fig. 4F). As expected, HK2 depletion also successfully abrogated the stimulatory effects on glycolysis rate, ATP levels, cell migration and colony formation in UBR7-deficient HepG2 cells (Fig. 4G-I). In agreement with previous findings, downregulation of HK2 expression fully abolished the proliferative and glycolytic phenotype in BEL-7404 cells transfected with UBR7 and HK2 shRNAs (Supplementary Fig. 6). Furthermore, coincident observations were achieved by xenograft model, showing that the promoting effects of UBR7 decline on tumor growth and metastasis was impaired by silencing HK2 (Fig. 4J). Taken as a whole, these observations indicate that HK2 is indispensable for regulation of glucose metabolism and tumorigenesis by UBR7 in HCC.

### UBR7 promotes Keap1 expression by ubiquitinating H2BK120ub on Keap1

**Fig. 5:**
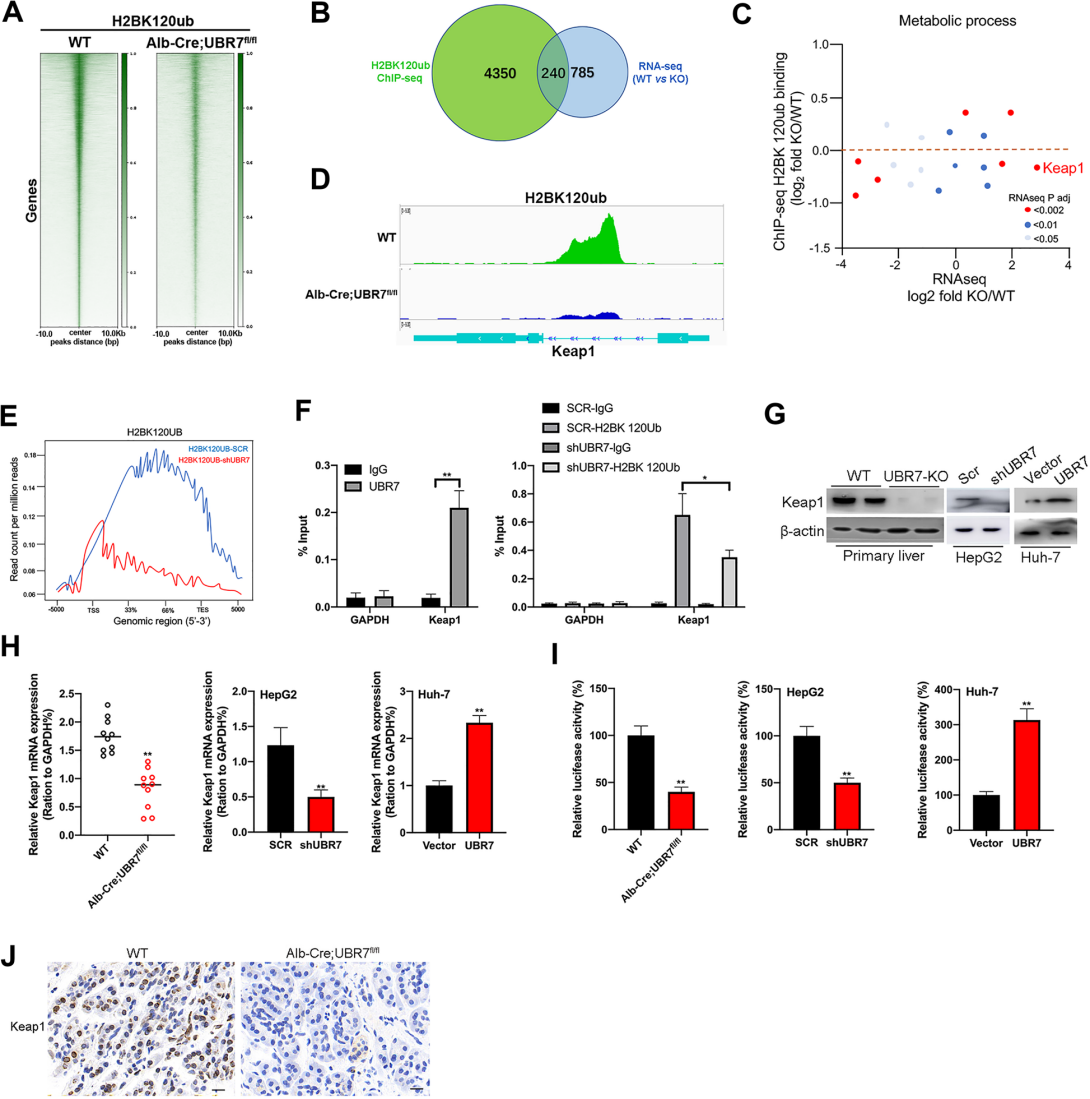
UBR7 promotes Keap1 expression levels by ubiquitinating H2BK120ub on Keap1. **A** Heatmap summarising chromatin immunoprecipitation (ChIP)-seq data for H2BK120ub, comparing liver cells from WT and Alb-Cre;UBR7^fl/fl^ mice. **B** The Venn diagram shows that the 240 genes combined with H2BK120ub in the ChIP-seq analysis and the genes regulated in the RNA-seq analysis overlap. **C** Diagram of metabolic process genes. x-axis, gene expression from RNA-seq data; y-axis, H2BK120UB binding level determined in ChIP-seq data. **D** Keap1 gene peak in liver cells from WT and Alb-Cre;UBR7^fl/fl^ mice. **E** The average gene density map of H2BK120Ub binding sites in Control (SCR) and UBR7-shRNA-expressing Huh-7 cells. **F** Left: Bar plot for quantitative PCR (qPCR) enrichment of UBR7 chromatin immunoprecipitation (ChIP) in Huh-7 cells for Keap1. GAPDH was used as a negative control. Right: Bar graph of qPCR enrichment of H2BK120Ub ChIP in Huh-7 cells expressing SCR or UBR7-shRNA. GAPDH was used as a negative control. **G**, **H**) Keap1 protein or mRNA expression levels in UBR7 KO primary hepatocytes and HepG2 cells, and Huh-7 cells overexpressing UBR7. **I** Fluorescein reporter gene analysis UBR7 bound to H2B on Keap1 in Alb-Cre;Ubr7^fl/fl^ primary hepatocytes and HepG2 cells, and Huh-7 cells overexpressing UBR7. **J** The expression level of Keap1 in WT and Alb-Cre;UBR7^fl/fl^ mice liver tissue was detected by immunohistochemistry. Data are shown as mean ± SD of three independent experiments. *p < 0.05, **p < 0.01. The scale bar in **J** was 100 μm

UBR7 has been reported to modulate the ubiquitination patterns of histone H2B at lysine120 (H2BK120ub) and serve as a tumor suppressor in breast cancer [9]. HK2 is not a direct substrate of UBR7, and the effect of UBR7 on lactate acid levels depends on its E3 ubiquitin ligase activity toward H2B monoubiquitination. Therefore, we analyzed the impact of UBR7-mediated H2BK120ub on the chromatin landscape. To do so, we immunoprecipitated chromatin bound to H2BK120ub in hepatocytes isolated from WT and Alb-Cre; UBR7^fl/fl^ mice and sequenced the associated DNA (Fig. 5A). A total of 4350 genes were found to be associated with H2BK120ub in this assay. By integrating chromatin immunoprecipitation sequencing (ChIP-seq) and the above RNA-seq data, we found that H2BK120ub bound to 240 differentially expressed genes in UBR7-deficient background compared to WT background (Fig. 5B). Among the ‘‘metabolic process’’ genes that showed lower H2BK120ub binding in UBR-7 silencing than WT cells, *Keap1* was the most significantly upregulated in the RNA-seq analysis (Fig. 5C). Analysis of enriched loci (peaks) in primary liver cells from WT and Alb-Cre; UBR7^fl/fl^ mice further indicated that overall H2BK120ub signals were drastically lower on the promoter region of *Keap1* in hepatocytes from Alb-Cre; UBR7^fl/fl^ mice **(**Fig. 5D), suggesting histone monoubiquitination is associated with activation of this promoter. Consistently, the intensity of H2BK120ub was remarkably reduced in UBR7-deficient Huh-7 cells as exhibited by average density plots (Fig. 5E), which was further confirmed by ChIP-qPCR analysis (Fig. 5F). Apparently, UBR7 could bind to *Keap1* promoter, and UBR7 silencing significantly reduced H2BK120ub binding to the promoter region of *Keap1* in Huh-7 cells. Coincident observations were also obtained by Immunoblotting assay, IHC staining, and qPCR analysis, demonstrating reduced Keap1 expression in UBR7-KO primary liver cells and UBR7-deficient HepG2 and BEL-7404 cells. Meanwhile, elevated Keap1 expression was found in Huh-7 and BEL-7402 cells with ectopic expression of UBR7 (Fig. 5G, H, J, Supplementary Fig. 7A, 7B). We next performed luciferase report assay to verify the participation of UBR7 in transcriptional activation of *Keap1*. Luciferase activity from the *Keap1* promoter was significantly lower in UBR7-KO primary liver cells, UBR7-deficient HepG2 and BEL-7404 cells, whereas the Luciferase signal was dramatically increased by UBR7 overexpression in Huh-7 and BEL-7402 cells (Figs. 5I, Supplementary Fig. 7C, 7D).

To further characterize the role of UBR7 in regulation of Keap1 expression, full length UBR-WT and UBR-CM were transfected into Huh-7 and BEL-7402 cells. ChIP-qPCR analysis showed that UBR7-CM overexpression essentially abolished binding to the promoter region of Keap1 in Huh-7 cells, which is consistent with the observation that UBR7-CM failed to promote H2B ubiquitination and Keap1 expression (Supplementary Fig. 8A-C). Similarly, UBR7-WT, but not UBR7-CM, increased the luciferase activity on the *Keap1* promoter in Huh-7 and BEL-7402 cells (Supplementary Fig. 8D-F). These results altogether suggest that UBR7 probably upregulates Keap1 expression levels by promoting monoubiquitination of histone H2B at lysine120, which affects binding of H2BK120ub on *Keap1* promoter region and subsequent transcription activation.

### Keap1 activation and Nrf2/Bach1 pathway inhibition are required for the UBR7-induced HK2 and glycolysis suppression

Keap1 is known as principal negative regulator of NF-E2-related factor 2 (Nrf2), a master transcription mediator of anti-oxidative responses as well as glycolysis [20, 21]. Keap1 loss and Nrf2 activation promotes lung cancer metastasis by accumulating BTB domain and CNC homolog 1 (Bach1), which has been reported to stimulate transcription of HK2 triggering glycolysis-induced metastasis [22, 23]. Consistently, Keap1 deficiency resulted in elevated expression of HK2, whereas Nrf2 knockdown decreased HK2 expression in Huh-7 cells (Supplementary Fig. 8G, 8H).

**Fig. 6:**
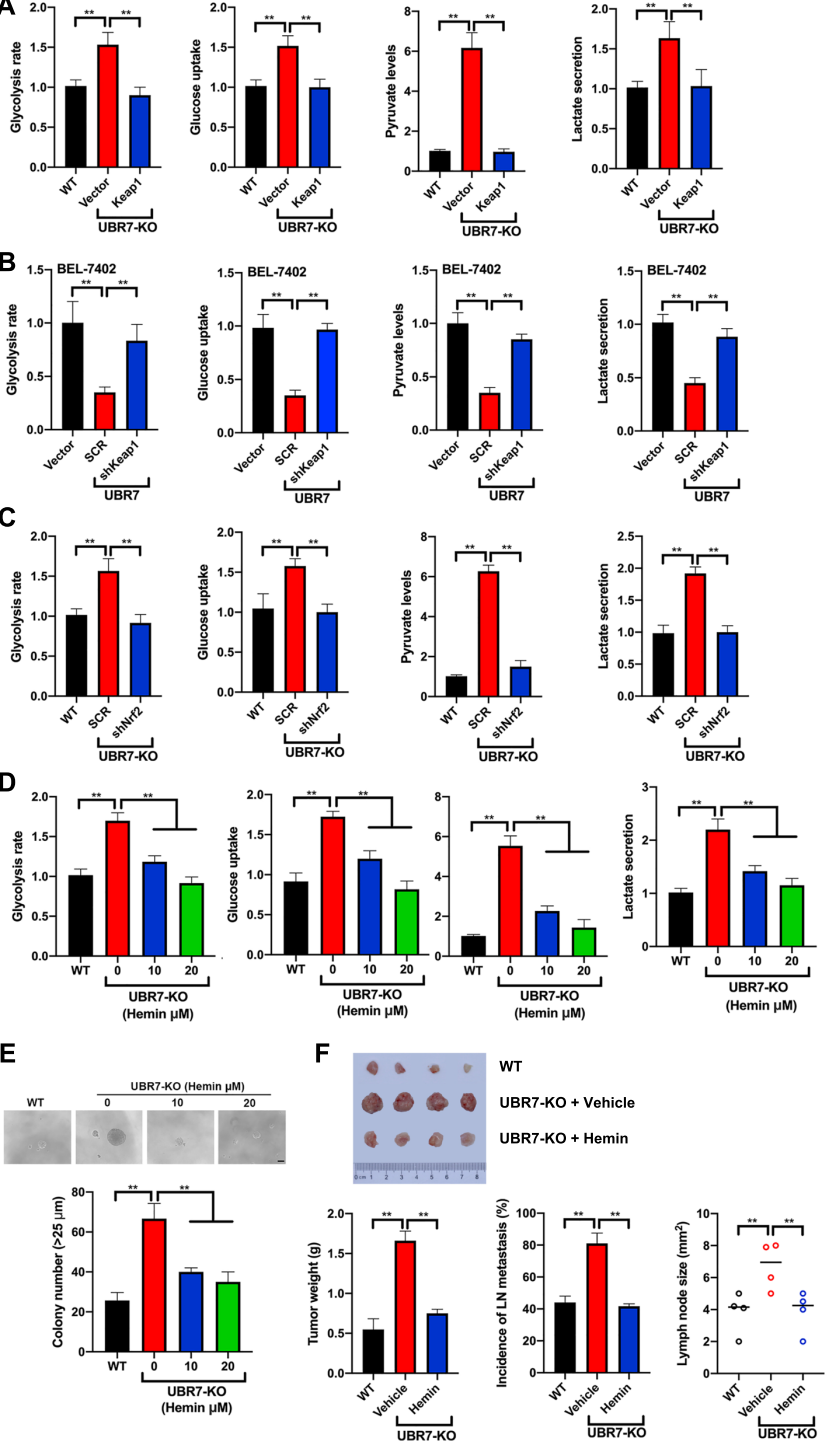
Keap1 activation and Nrf2/Bach1 pathway inhibition are required for the UBR7-induced HK2 and glycolysis suppression. **A** The levels of glycolysis, glucose uptake, pyruvate and lactate secretion in UBR7-KO cells overexpressing Keap1. **B** The levels of glycolysis, glucose uptake, and pyruvate and lactate secretion in BEL-7402 cells overexpressed UBR7 and knocked out Keap1. **C** The levels of glycolysis, glucose uptake, pyruvate and lactate secretion in UBR7-KO cells knocked out Nrf2. **D** The levels of glycolysis, glucose uptake, pyruvate and lactate secretion in UBR7-KO cells treated with Hemin. **E** The levels of colony number in UBR7-KO cells treated with Hemin. **F** UBR7-KO cells treated with Hemin were injected subcutaneously into nude mice to analyse xenogeneic tumours, tumour weight, lymph node metastasis and lymph node size levels. Data are shown as mean ± SD of three independent experiments. **p < 0.01. The scale bar in (**E**) was 100 μm

Both our ChIP-seq and RNA-seq data revealed that Keap1 was enriched in metabolic process network, and HK2 is the major glycolytic enzyme, therefore, we decided to test whether Keap1-Nrf2 axis was involved in UBR7-induced glycolysis suppression. For this purpose, vectors with or without Keap1 insert were transfected into UBR7-KO primary liver cells and BEL-7404 cells to evaluate the glycolytic phenotypes. Of note, forced expression of Keap1 almost completely abrogated the glycolysis rate, glucose uptake, pyruvate levels and lactate production elicited by UBR7 deletion (Fig. 6A, Supplementary Fig. 9A-D). Meanwhile, Keap1 overexpression successfully abolished the induction of Nrf2, Bach1 and HK2 expression by UBR7 deletion, highlighting the critical role of Keap1 in this pathway (Supplementary Figs. 10A, 10B). Conversely, downregulation of Keap1 substantially rescued the glycolytic phenotypes in BEL-7402 cells overexpressing UBR7 (Fig. 6B). Concordant results were observed in immunoblotting assay, displaying that Keap1 silencing reversed the protein levels of downstream genes elicited by UBR7 overexpression (Supplementary Fig. 10C).

Given the well-established interplay between Keap1 and Nrf2, we next set out to determine whether Nrf2 is also implicated in UBR7-induced glycolysis suppression. To this end, we silenced Nrf2 in the UBR7-KO primary liver cells and BEL-7404 cells to evaluate the glycolytic phenotypes as well as downstream proteins. As expected, Nrf2 depletion completely abolished the glycolytic phenotypes and downstream proteins elicited by UBR7 knockout (Fig. 6C, Supplementary Fig. 10D, 11).

Bach1 functions as a molecular sensor and effector of heme, which could be inhibited by addition of hemin, a heme derivative[24]. Therefore, we first detected whether UBR7 could regulate the expression of Bach1 in Huh-7 cells. UBR7 silencing increased the expression of Bach1 while UBR7 overexpression downregulated the expression of Bach1 (Supplementary Fig. 10E, 10F). Then we treated the UBR-7 KO primary liver cells with hemin to test if Bach1 degradation would affect the HK2 expression and glycolysis. (Fig. 6D, Supplementary Fig. 10G). As expected, Hemin dramatically reduced the protein levels of Bach1 and HK2 with no alterations on Keap1 and Nrf2. Besides, Bach1 inhibition with Hemin significantly prevented the glycolysis induced by UBR7 knockout, suggesting the involvement of Bach1 in Keap1-Nrf2-mediated glycolysis and HK2 expression. Given the importance of HK2 in glucose metabolism and tumorigenesis inhibited by UBR7 in HCC, we next examined the role of its regulator Bach1 in UBR7-mediated HCC development. Notably, Hemin treatment almost fully abolished the effects of UBR7 depletion on colony formation, as well as tumor weight, lymph node metastasis, and lymph node size (Fig. 6E, F). Taken together, these data signified that Keap1 activation and Nrf2/Bach1 inhibition are essential for UBR7-induced suppression on HK2 and glycolysis.

### Glycolysis inhibition prevents UBR7 deficiency-induced tumor growth

To further characterize the connection between UBR7-mediated glycolysis and tumor growth in HCC, we next evaluated whether drugs, which target enzymes involved in glycolysis, could affect UBR7-deficiency induced tumorigenesis (Supplementary Fig. 12A). In response to HK2 inhibitors, such as 2-deoxyglucose (2-DG) and lonidamine (LND), UBR-7 KO primary liver cells exhibited about 50% lower viability and colony formation ability, showing greater dependence on glycolysis than WT cells. Inhibiting lactate secretion with the MCT-1 inhibitor AZD3965, also greatly reduced viability and colony formation ability in UBR7- KO cells. However, this inhibitory effect was not observed in response to dicholoroacetate (DCA), a pyruvate dehydrogenase kinase (PDK) inhibitor that inhibits lactate production (Supplementary Fig. 12B-E). Intriguingly, synergistic inhibition of viability and colony formation by UBR7 overexpression and glycolysis inhibitors was observed in BEL-7402 cells, suggesting the existence of other potential mediating mechanism (Supplementary Fig. 13 and 14). In vivo evidence further highlighted the importance of glycolysis in UBR7 deficiency-induced tumor growth as glycolysis inhibitors completely abolished the promoting effect elicited by silencing UBR7 (Supplementary Fig. 12F).

### ALKBH5-mediated m6A modification leads to dysregulation of Ubr7 expression.

**Fig. 7:**
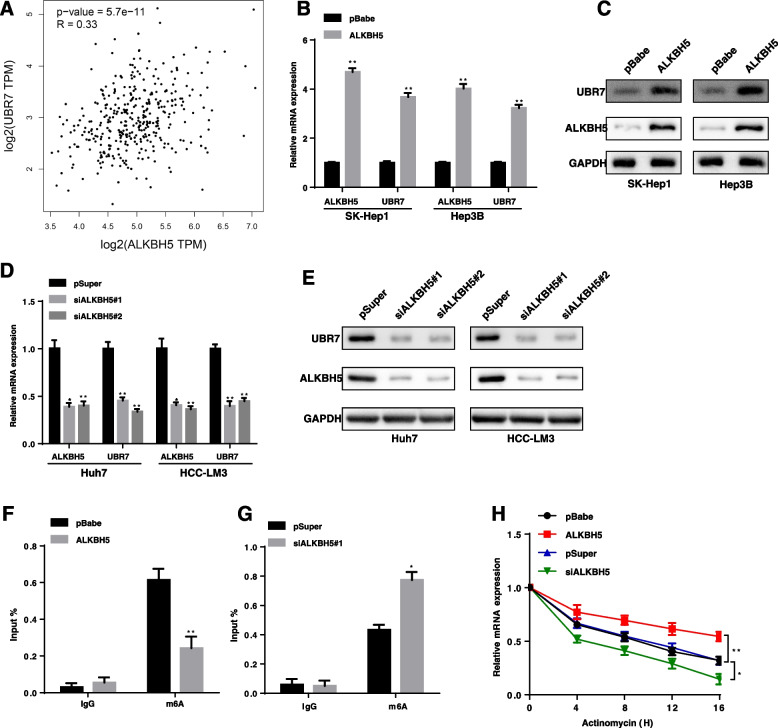
ALKBH5 regulates UBR7 expression through m6A modification. **A** UBR7 expression was positively correlated with ALKBH5 expression in an analysis of TCGA data. **B-E** qRT-PCR and western blot analysis of ALBKH5 and UBR7 upon ALKBH5 silencing or overexpression. **F, G** RIP-qPCR showed the enrichment of UBR7 m^6^A upon ALKBH5 silencing or overexpression. **H** The decay rate of UBR7 mRNA in cells treated with 2.5 mmol/L actinomycin D for indicated times upon ALKBH5 silencing or overexpression. Data are shown as the mean ± SD. ^*^P < 0.05; ^**^P < 0.01

To explore the upstream regulation mechanism of Ubr7 downregulation in HCC, we found numerous m^6^A modification sites are distributed in UBR7’s mRNA from RMBase database. RNA N6-methyladenosine (m^6^A) modification is widely involved in the metabolic reprogramming through regulating mRNA stability in tumour cancer [25]. ALKBH5 is one of the key members of the m6A demethylases and was also found to be also downregulated in HCC [26]. Interestingly, we observed that a significant positive correlation between ALKBH5 and UBR7 protein level in the Cancer Genome Atlas (TCGA) database of HCC patients (Fig. 7A). Therefore, we hypothesized that UBR7 expression is dependent on m6A modification that is regulated by ALKBH5. Indeed, compared to the control, ALKBH5 overexpression significantly increased UBR7 mRNA level and protein level in HCC cell lines (Fig. 7B, C), while ALKBH5 silencing had the reverse effect (Fig. 7D, E). Consistently, m6A measurement by using RNA-binding protein immunoprecipitation (RIP) assays showed that ALKBH5 overexpression increased m6A level of UBR7’s mRNA in HCC cells (Fig. 7F). In contrast, ALKBH5 silencing could significantly reduce m6A modification of UBR7 mRNA (Fig. 7G). We further assessed the UBR7 mRNA stability upon ALKBH6 depletion or overexpression. After treating cells with actinomycin D in order to block the de novo synthesis of RNA, ALKBH5 depletion resulted in a decreased stability of UBR7 mRNA, whereas overexpression of ALKBH5 could increase the stability of UBR7 mRNA (Fig. 7H). Together, these data reveal that ALKBH5 stabilizes Ubr7 mRNA through m6A modification and regulates its expression level.

### Clinical significance of UBR7 and the Keap1/Nrf2/Bach1/HK2 axis in human HCC tissue.

**Fig. 8:**
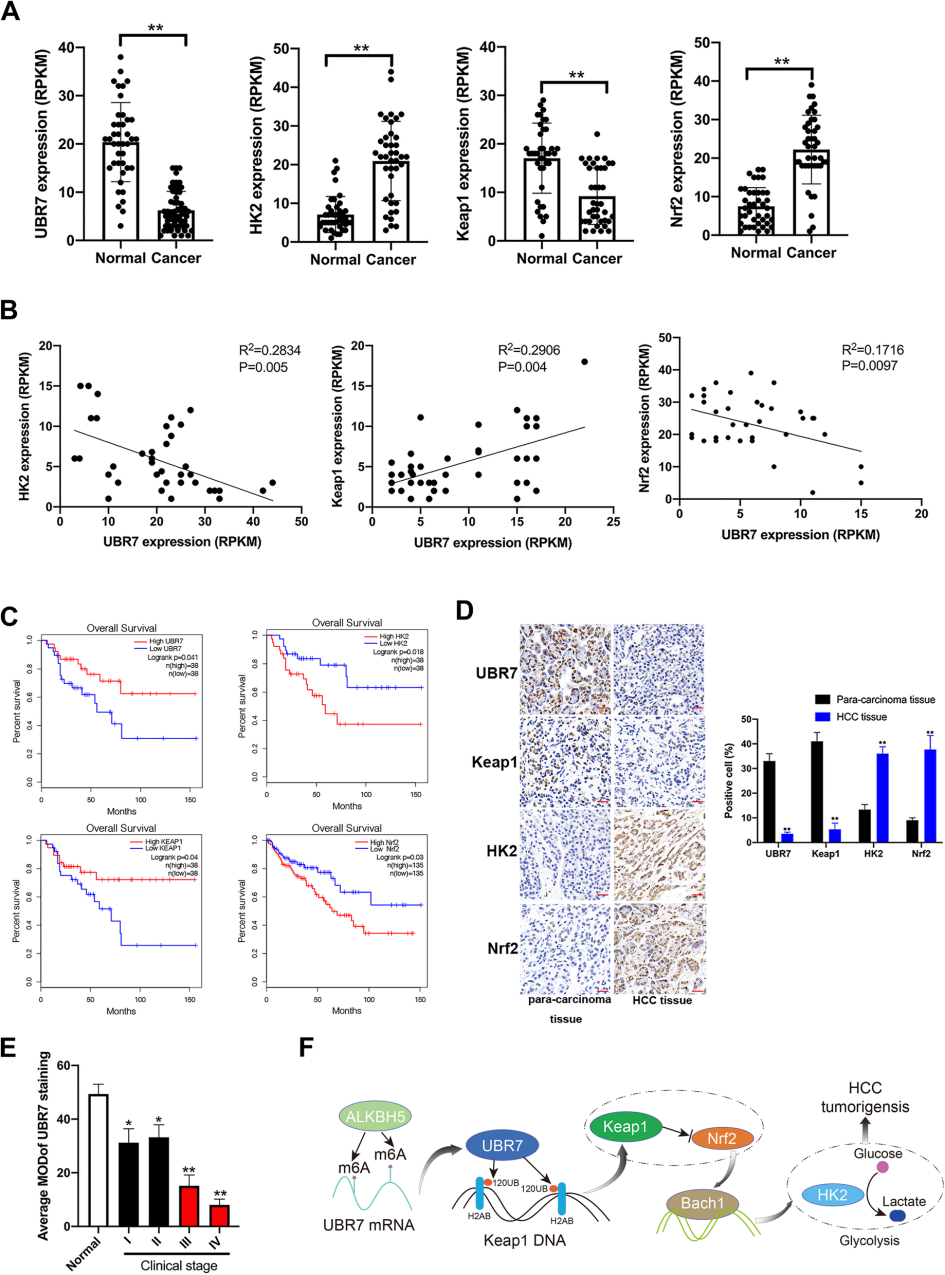
Clinical significance of UBR7 and its association with the Keap1/Nrf2/BACH1/HK2 axis in human HCC tissue. **A** TCGA data analysed the expression level of UBR7, Keap1, Nrf2 and HK2 in hepatocellular carcinoma. **B** The TCGA database analysed the relationship between the expression levels of UBR7, Nrf2 and HK2. **C** The survival of patients with hepatocellular carcinoma. **D** The expression levels of UBR7, Keap1, Nrf2 and HK2 in samples from patients with hepatocellular carcinoma. **E** Relationship between UBR7 and clinical stage of hepatocellular carcinoma. **F** Schematic diagram of UBR7 negatively regulating HK2-mediated glycolysis. *p < 0.05, **p < 0.01

To reveal the clinical relevance of UBR7 with Keap1/Nrf2/HK2 axis in HCC, TCGA data of these genes were analyzed. In contrast to normal adjacent tissues, UBR7 and Keap1 mRNA levels were found to be notably downregulated in HCC tissues, whereas HK2 and Nrf2 were upregulated (Fig. 8A). UBR7 and HK2 expression levels were negatively correlated in HCC tissues, as were UBR7 and Nrf2. Conversely, Keap1 was positively correlated with UBR7 in HCC, as the negative regulator of Nrf2 (Fig. 8B). In line with these observations, low levels of UBR7 and Keap1 predicted poor clinical outcome in HCC, whereas high levels of Nrf2 and HK2 correlated with shorter survival of patients (Fig. 8C). These TCGA data was further confirmed by our immunohistochemical analysis of clinical specimens (Fig. 8D). Indeed, the expression level of UBR7 was positively correlated with Keap1 expression in these clinical species, but it was negatively correlated with HK2 and Nrf2 expression (Supplementary Fig. 15). Besides, the protein levels of UBR7 was significantly lower in HCC tissue, which differed with the stage of tumor development (Fig. 8E). Clinicopathological analysis was further constructed to demonstrate the clinical significance of UBR7, especially in terms of tumor diameter and differentiation (Supplementary Table 1). Overall, the results derived from human datasets and clinical samples strongly supported the possibility that UBR7 act as a tumor suppressor in HCC by regulating Keap1/Nrf2/Bach1/HK2 axis (Fig. 8F).

## Discussion

Due to their high substrate specificity, E3 ubiquitin ligase machineries have emerged as attractive therapeutic targets for cancer treatment. Recent years have seen the advent of molecular glues or PROTAC degraders that hijack E3 catalytic activity to specifically target and degrade intracellular disease-driving proteins, which highlights the importance to understand the substrate-bound structures and cellular functions of E3 ligases [27]. Among the seven UBR family members in human, UBR7 is the most divergent one that act specifically within the N-end rule pathway and possesses the PHD finger with similar patterns of Cys and His residue to that of the RING domain [28]. In this study, we report the protective role of UBR7 on HCC carcinogenesis by preventing metabolic reprogramming toward aerobic glycolysis. Mechanistically, UBR7 impedes aerobic glycolysis by mediating transcriptional activation of Keap1 and its downstream Nrf2/Bach1/HK2 signaling via monoubiquitinating histone H2B.

UBR box E3 ligases like UBR1, UBR2, UBR4, and UBR5 are the primary N-recognins, which have a wide variety of physiological substrates and play essential regulatory roles in many signaling pathways, such as apoptosis, G-protein signaling, inflammation, mitochondrial quality control, and replication stress, in well-characterized mechanisms [8]. The link between UBR family and glycolysis is elusive, although UBR5 has been reported to promote aerobic glycolysis in PDAC and promote tumor growth [29]. UBR3, UBR6 and UBR7 don’t bind to N-degrons, and their functions remain largely unknown. In this study, however, we identified UBR7’s inhibitory role in glycolysis for the first time in MHCC-97L cells by our functional screening assay in UBR7-silenced HCC cells. Of note, the effect of UBR7 on lactate acid levels relied on its E3 ubiquitin ligase activity toward H2BK120ub, as supported by the null inhibition of UBR7 catalytic mutant of H163S/H166S.

Growing evidence has highlighted the importance of monoubiquitination as one of the largest histone post-translational modifications. Unlike polyubiquitination, which marks the protein for degradation via the proteasome, monoubiquitination refers to the covalent attachment of a single ubiquitin to specific lysines of histone tails. This post-translational modification on histones was involved in many fundamental cellular processes including transcriptional elongation, DNA damage response, stem cell differentiation and oncogenesis [30, 31]. Monoubiquitination at lysine 120 on histone H2B has tumor suppressive roles as global levels of H2BK120ub are low to absent in advanced cancers. Moreover, classic tumor suppressors, such as p53, BRCA1 or RING finger E3 ubiquitin ligases RNF20 and RNF40, are reported as partners of H2BK120ub interactomes [32]. UBR7 downregulation in HCC patients was dependent on the ALKBH5 mediated m6A modification, which could affect mRNA stability. Consistently, UBR7 loss was observed in both GISTIC database and primary liver tumor samples, suggesting a putative tumor suppressive function. To address this possibility, gain- and loss-of-function assays were performed in HCC cell lines and corresponding xenografts, and liver-specific UBR7 knockout mice (Alb-Cre; UBR7^fl/fl^ mice) exhibited much faster disease progression, indicating the inhibitory effect of UBR7 on HCC tumorigenesis. Similar observations have been found in breast cancer that UBR7 acts as an H2BK120ub ligase and a tumor suppressor[9]. Besides, manumycin polyketide natural products (asukamycin and manumycin A) function as molecular glue between UBR7 and p53 to transactivate p53 and suppress breast cancer [33]. Apparently, aggregated knowledge of H2BK120ub and its interactome like UBR7 might confer new opportunities for therapeutic targeting of malignancy. Despite the anti-cancer function in solid tumors like breast cancer and HCC, UBR7 was identified as transcriptional target of NOTCH1 and played an oncogenic role in T cell acute lymphoblastic leukemia [12]. Thus, further investigation is needed to fully elucidate the conflicting role of UBR7 in cancer.

H2BK120ub has been shown to promote local chromatin relaxation, fostering a more open chromatin structure accessible to transcription factors. Here we demonstrated that Keap1 is a major target downstream of UBR7, and the enrichment of H2BK120ub on its promoter is mediated by UBR7. UBR7-mediated H2BK120ub enrichment boosted the transcriptional activation of Keap1, restraining the downstream Nrf2/Bach1/HK2 signaling. Besides, H2BK120 ubiquitination has been reported to play central roles in histone cross-talk, affecting methylation events on histone H3 by recruiting methyltransferase complexes, which includes H3K4 di- and tri-methylation (H3K4me3), as well as H3K79 tri-methylation (H3K79me3) [32]. Epigenetic modifications, such as enrichment of H3K4Me3 and H3K27Ac on the promoter of KEAP1, was known to regulate KEAP1 expression in colorectal cancer [34]. Furthermore, UBR7 binds to post-nucleosomal H3K4me3 and H3K9me3 histones via its UBR box and PHD finger, triggering reincorporation of post-nucleosomal H3 complexes as a histone chaperone [11]. UBR7 depletion significantly reduced H3K79Me2, which was primarily present in conjunction with H2BK120ub [9]. Therefore, functional crosstalk between H2B ubiquitylation at Lys120 (H2BK120ub) and H3 methylation under regulation of UBR7 on Keap1 expression requires further exploration, which may aid epigenetic drug development in the future.

## Conclusion

Our study illustrates that UBR7 inhibits HCC tumorigenesis via targeting glycolysis, the protective role of which is dependent on its catalytic activity toward H2BK120ub. H2BK120ub probably forms a loose chromatin structure to induce the transcription of Keap1 which negatively regulate the downstream Nrf2/Bach1/HK2 signaling. Pharmaceutical and genetic inhibition of glycolytic enzymes diminishes the stimulatory effect of UBR7 deficiency on tumor growth. The reduced level of UBR7 observed in HCC resulted from its decreased mRNA stability, which was mediated by ALKBH5-dependent m6A modification of specific adenosines in UBR7. Our findings raise the possibility that targeting glycolysis to UBR7 in HCC might be a potential therapeutic strategy.

## Supplementary Information

**Additional file 1.**

## Data Availability

ChIP-Seq and RNA-Seq data can be accessed at GEO with the accession number GSE173454 and GSE173501. All relevant data can be obtained from the author upon request.

## Abbreviations

UBR7: Ubiquitin protein ligase E3 component N-recognin 7
PHD: Plant homeodomain
H2BK120ub: Monoubiquitination of histone H2B at lysine 120
HCC: Hepatocellular carcinoma
UBR: Ubiquitin protein ligase E3 component N-recognin
HK: Hexokinase
shRNA: Small hairpin RNA
siRNAs: Small interfering RNAs
UBR7-WT: BEL-7402 cells expressing wild-type
UBR7-CM: H163S/H166S catalytic-mutant
RNA-seq: RNA-sequencing
GO: Gene Ontology
GSEA: Gene Set Enrichment Analysis
OCR: Oxygen consumption rate
ChIP-seq: Chromatin immunoprecipitation sequencing
Nrf2: NF-E2-related factor 2
Bach1: BTB domain and CNC homolog 1
2-DG: 2-Deoxyglucose
LND: Lonidamine
DCA: Dicholoroacetate
PDK: Pyruvate dehydrogenase kinase
TCGA: The Cancer Genome Atlas
RIP: RNA-binding protein immunoprecipitation
H3K4me3: H3K4 di- and tri-methylation
H3K79me3: H3K79 tri-methylation
