# Supplementary material for: UBR7 inhibits HCC tumorigenesis by targeting Keap1/Nrf2/Bach1/HK2 and glycolysis
Source: J Exp Clin Cancer Res. 2022 Nov 24;41:330. doi: 10.1186/s13046-022-02528-6 (PMC9686014; doi:10.1186/s13046-022-02528-6)
Supplement: Supplementary file 1 — Additional file 1. [file 13046_2022_2528_MOESM1_ESM.docx]

**Supplementary methods:**

**Antibodies and Reagent**

UBR7 antibody (ab228911), HK2 antibody (ab209847), Keap1 antibody (ab227828), Nrf2 antibody (ab137550) and Bach1 antibody (ab180853) were purchased form Abcam (Cambridge, United Kingdom). Hemin (S5645), 2-Deoxy-D-glucose (2-DG, S4701), lonidamine (LND, S2610), sodium dichloroacetate (DCA, S8615) and AZD3965 (S7339) were purchased from Selleck.

**Western bolt**

Cells were lysed with RIPA lysate (Beyotime Biotechnology, Beijing, China) containing protease inhibitors, and the protein supernatant was collected; an appropriate amount of protein loading buffer was added to the protein supernatant and boiled for 5 minutes to denature the protein. The proteins were electrophoresed sequentially, transferred and blocked, then the corresponding primary antibodies were added and incubated at 4°C overnight. The corresponding secondary antibody was added and incubation was at room temperature for 1 hour. Exposure was performed using BioImaging Systems instruments to obtain pictures of protein expression.

**Immunohistochemistry**

Paraffin sections and tumor tissue of patients with hepatocellular carcinoma were successively dewaxed, repaired with citric acid antigen, and treated with 3% hydrogen peroxide, followed by serum blocking and antibody incubation. The source of patient tissue specimens has been described in the supplementary table. Finally, the Image-Pro software was used to analyse the positive or negative regions of tissue sections for analysis. The labeling index percentages of UBR7, HK2, Keap1 and Nrf2 were determined by examining ≥1,000 tumor cells in random 3 high-powered fields. The expression levels of HK2, Keap1 and Nrf2 were divided into low expression (＜20% positive cells) and high expression (≥20% positive cells) groups. The expression level of UBR7 was divided into low expression (＜50% positive cells) and high expression (≥50% positive cells) groups

**qRT-PCR**

The total RNA of HCC cells was obtained by the Trizol method and reverse transcribed into cDNA. The subsequent qRT-PCR was performed according to the following primers: 5’- CCAGAACAGGGAAAGGATGA-3’ (UBR7 Forward) and 5’- TCCTGAAGTTTGCAGCCAG -3’ (UBR7 Reverse); 5’- CTCAACCGCTTGCTGTATGC-3’ (Keap1 Forward) and 5’- CTCTGTCTCCACGTCGTAGC-3’ (Keap1 Reverse).

**Chromatin Immunoprecipitation (ChIP) and sequencing**

Using the same primary liver cells as RNA-seq, the ChIP procedure follows the MagnaChIP kit guide (Millipore). The cells were fixed with 1% formaldehyde, and then incubated with H2BK120Ub antibody and rabbit IgG (Millipore); then, the chromatin was sheared and subjected to immunoprecipitation. Specific primers were used and the DNA was divided by qPCR. Then the DNA library was established and sent for sequencing. BWA aln (v0.7.5a) was used to compare the readings with the human genome hg19, and MACS 2.0 with q-value cut-off was used to evaluate the peak call. The peak call analysis of each ChIP-seq is implemented independently. We then created a specific peak list for each marker, including familiar peaks that were repeated at least twice. Annotation and pattern analyses were completed using HOMER (v4.7.2), p value <10^-30^. Use the integrated genomics viewer (IGV 2.3.34) for presentation. The intensity curve of ChIPseq data was completed using ngsplot version 2.61.

**Supplementary Table 1**

**Correlations between UBR7 expression and clinicopathologic features**

**in 47 pairs of HCC patients**

| Variable | Expression of UBR7 | | Total | *P*^a^ |
| --- | --- | --- | --- | --- |
|  | Low | High |  |  |
| Age (years)  ≤50  >50 | 13  20 | 6  8 | 19  28 | 0.825 |
| Gender  Male  Female | 19  11 | 10  7 | 29  18 | 0.760 |
| HBsAg  Negative  Positive | 12  21 | 5  9 | 17  30 | 0.966 |
| AFP  ≤ 20  > 20 | 13  17 | 7  10 | 20  27 | 0.886 |
| γ-GT (U/L)  ≤54  >54 | 15  17 | 7  8 | 22  25 | 0.989 |
| Liver cirrhosis  No  Yes | 11  21 | 5  10 | 16  31 | 0.944 |
| Tumor Diameter (cm)  ≤5  >5 | 21  9 | 6  11 | 27  20 | 0.021 |
| Tumor Differentiation  I+II  III+IV | 21  11 | 5  10 | 26  21 | 0.038 |
| TNM stage  I  II+III | 20  9 | 12  6 | 32  15 | 0.869 |

^a^*p*-value < 0.05 was considered statistically significant. *p*-values were calculated using the Pearson chi-square test.

**Supplementary Figures**


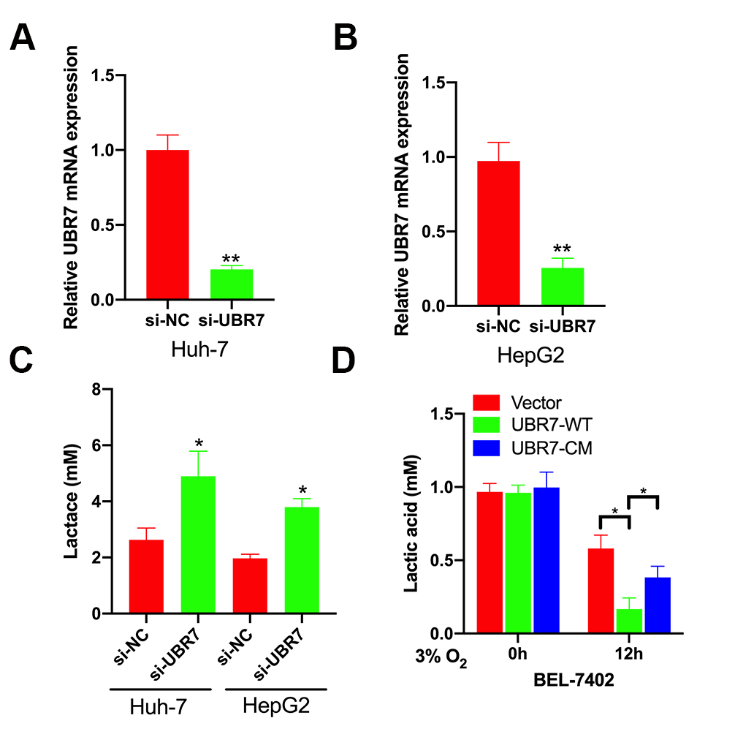


**Supplementary Fig. 1** **Effects of UBR7 on lactate secretion of HCC cells.** (**A**, **B**) UBR7 was knocked out in Huh-7 and HepG2 cells to detect the expression level of UBR7mRNA. (**C**) Lactate secretion levels in Huh-7 and HepG2 cells knocked out of UBR7. (**D**) BEL-7402 cells overexpress UBR7-WT or UBR7-CM (H163S/H166S catalytic-mutant) to detect lactate secretion. Data are shown as mean ± SD of three independent experiments. *p < 0.05, **p < 0.01.

**Supplementary**
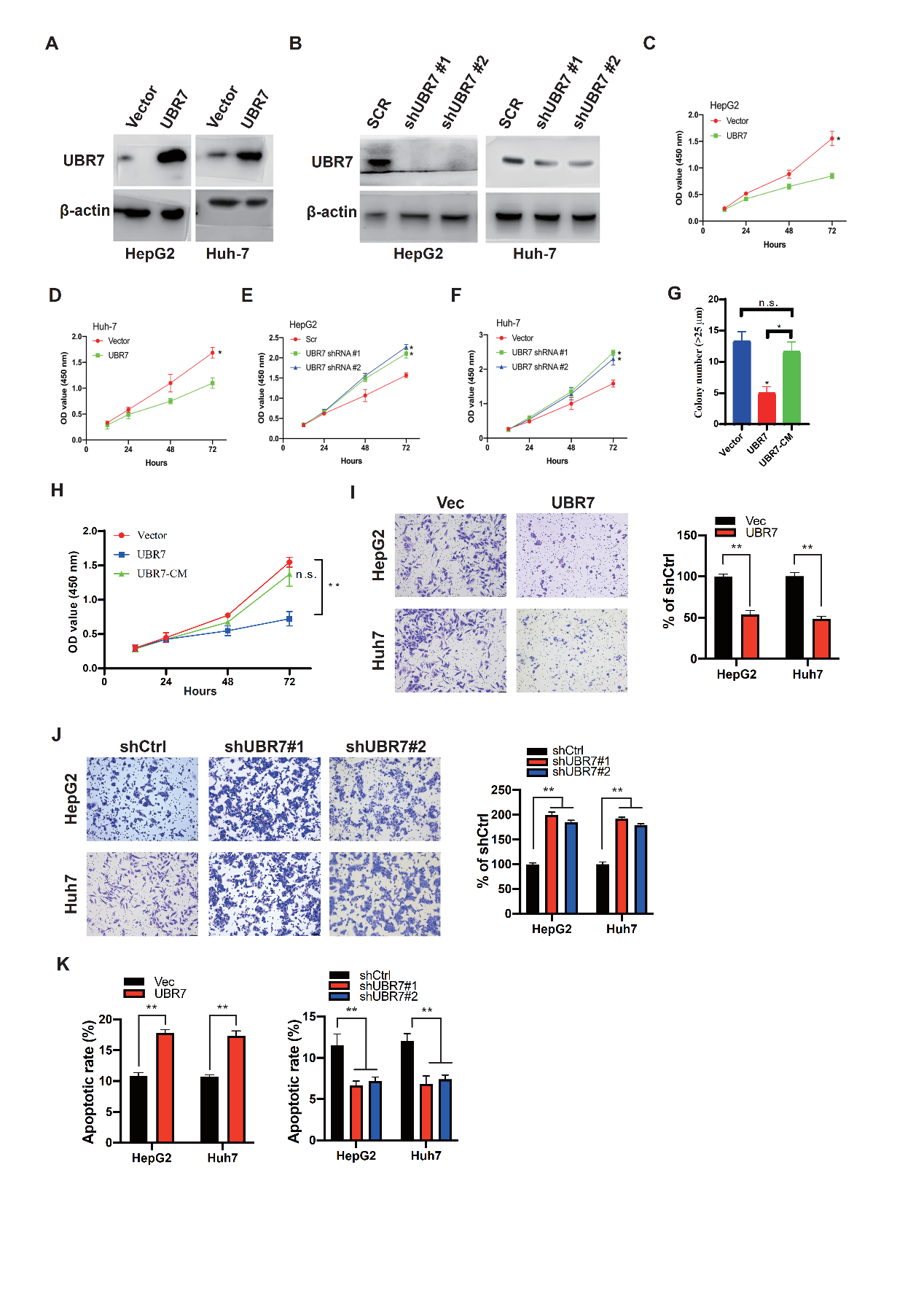
**Fig. 2** **The effect of UBR7 on cell viability of HCC cells.** (**A**, **B**) Western blot was used to detect the expression level of UBR7 in HepG2 and Huh-7 cells. (**C**, **D**) Cell viability of Huh-7 and HepG2 cells upon UBR7 overexpression. (**E**, **F**) Cell viability of Huh-7 and HepG2 cells knocked out UBR7. (**G**, **H**) The clonal sphere forming ability and cell viability of BEL-7402 cells overexpressing UBR7-WT or UBR7-CM. (**I, J**) Cell invasion of Huh-7 and HepG2 cells that were either overexpressing or silencing UBR7. (**K**) Cell apoptosis of Huh-7 and HepG2 cells when overexpressing or silencing UBR7. Data are shown as mean ± SD of three independent experiments. *p < 0.05, **p < 0.01, n.s. = no difference.


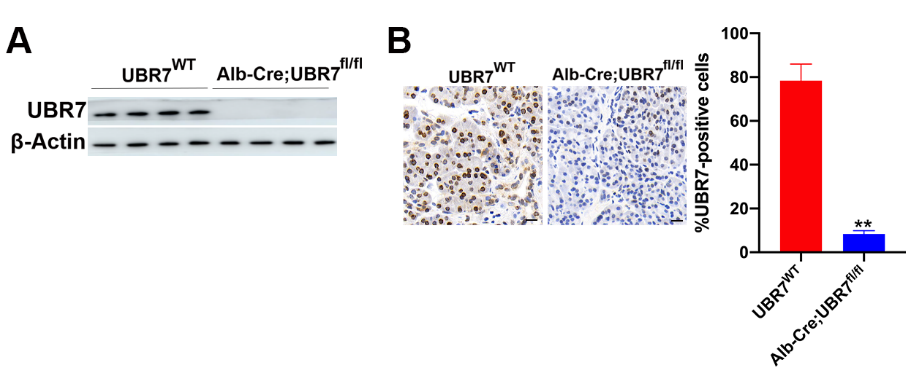


**Supplementary Fig. 3** **UBR7 protein expression level in Alb-Cre; UBR7^fl/fl^ mice**. Western blot (**A**) and immunohistochemical staining (**B**) were used to detect the expression of UBR7 in Alb-Cre; UBR7^fl/fl^ and UBR7^WT^ mice. The scale bar in B was 100μm.


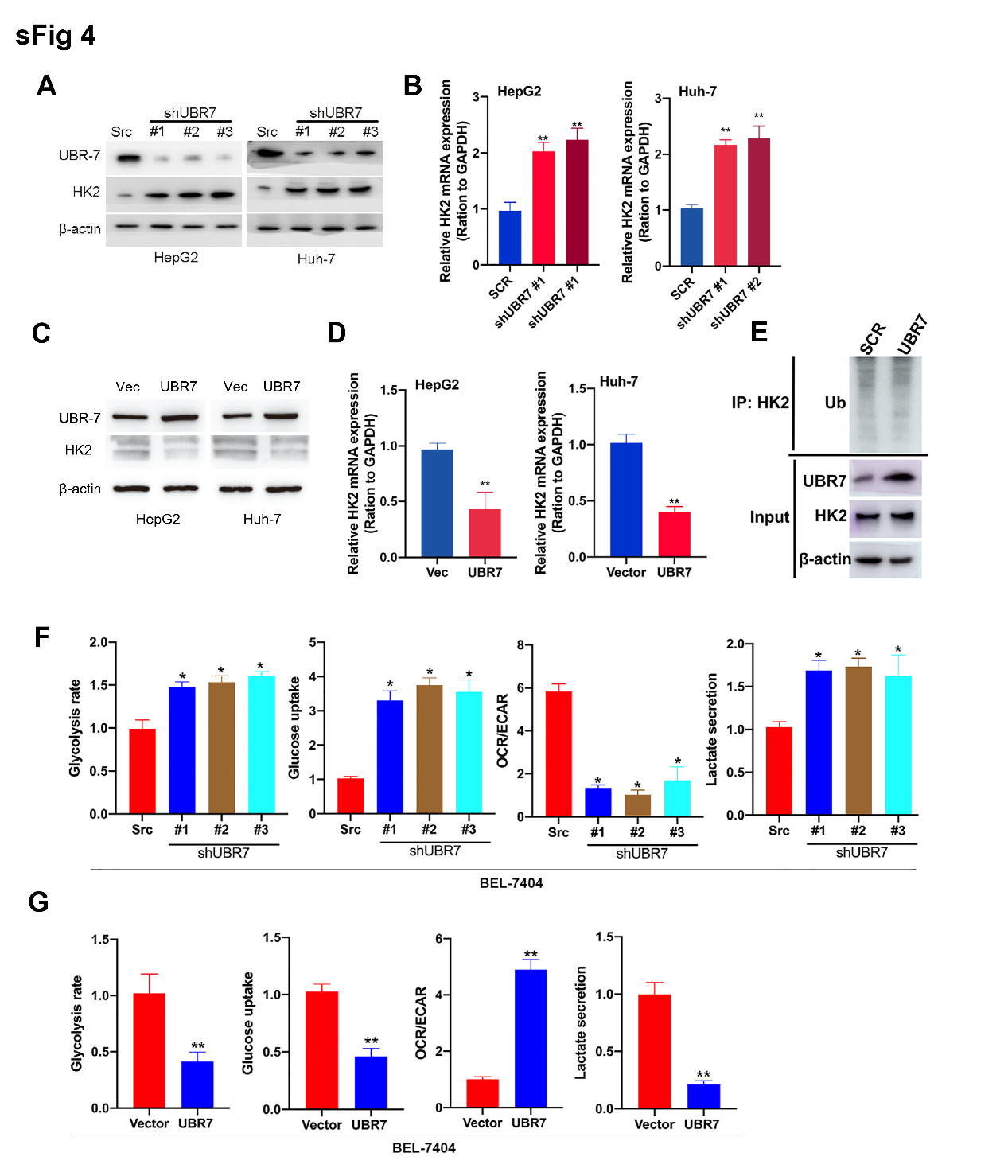


**Supplementary Fig. 4** **Effect of overexpression or knockout of UBR7 on glycolysis in HCC cells.** (**A**, **B**) HepG2 and Huh-7 cell lines were transiently transfected with shUBR7, and Western blot and qRT-PCR were used to detect HK2 protein and mRNA expression. (**C, D**) HepG2 and Huh-7 cells overexpressed UBR7 stably, and western blot and qRT-PCR were used to detect HK2 protein and mRNA expression. (**E**) Co-immunoprecipitation was used to detect the ubiquitination level of HK2 protein by UBR7. (**F**) Glycolysis, glucose uptake, OCR and lactate secretion levels in BEL-7404 cells knocked out UBR7. (**G**) Glycolysis, glucose uptake, OCR and lactate secretion levels in BEL-7404 cells overexpressing UBR7. Data are shown as mean ± SD of three independent experiments. *p < 0.05, **p < 0.01.


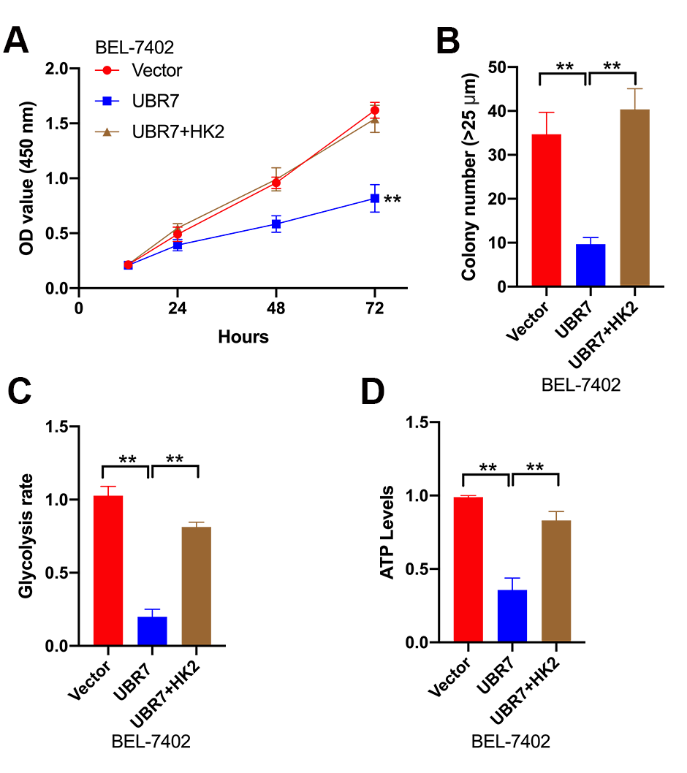


**Supplementary Fig. 5** **Effects of overexpression of UBR7 and HK2 on HCC cell function and glycolysis.** Cell viability (**A**), clonal formation (**B**), glycolysis rate (**C**) and ATP levels (**D**) in BEL-7402 cells overexpressing UBR7 and HK2. Data are shown as mean ± SD of three independent experiments. **p < 0.01.


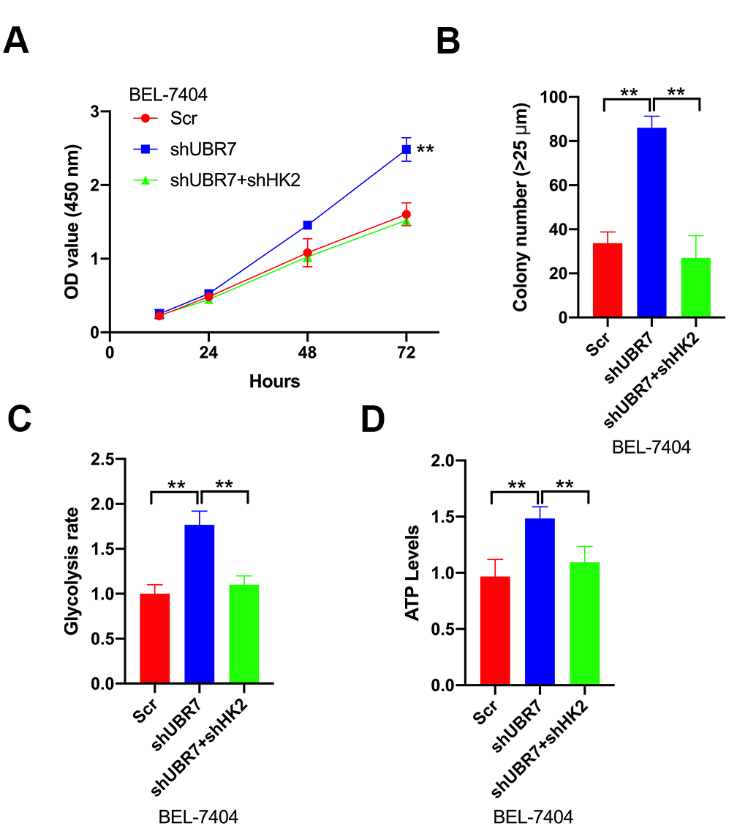


**Supplementary Fig. 6** **Knock out UBR7 and HK2 to detect the proliferation and glycolysis of HCC cells.** Cell viability (**A**), clonal formation (**B**), glycolysis rate (**C**) and ATP levels (**D**) in BEL-7404 cells knocked out of UBR7 and HK2. Data are shown as mean ± SD of three independent experiments. **p < 0.01.


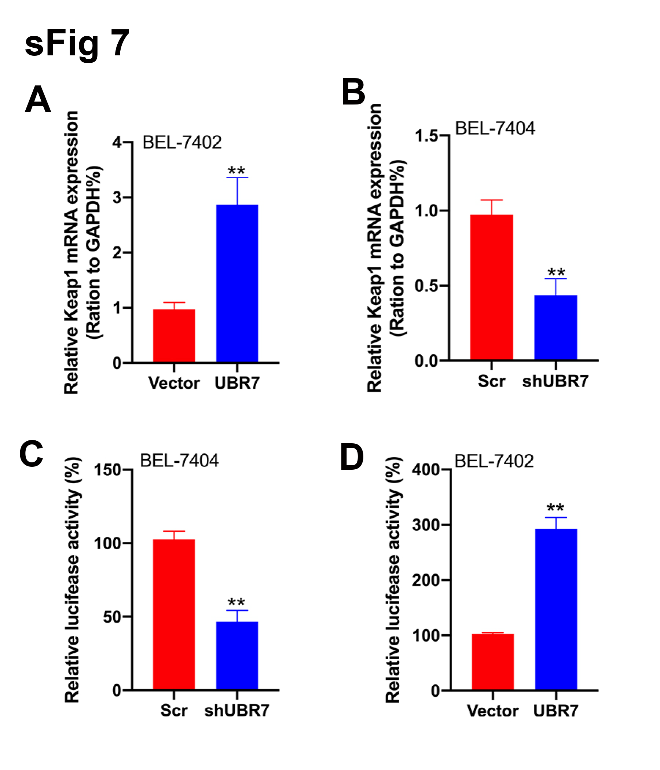


**Supplementary Fig. 7** **UBR7 promotes Keap1 expression.** (**A**, **B**) Overexpress or knock out UBR7 to detect Keap1 mRNA level. (**C**, **D**) Fluorescein reporter gene analysis UBR7 binds to H2B on Keap1 in BEL-7402 cells overexpressing UBR7 and BEL-7404 knocked out UBR7. Data are shown as mean ± SD of three independent experiments. **p < 0.01.

**Supplementary**
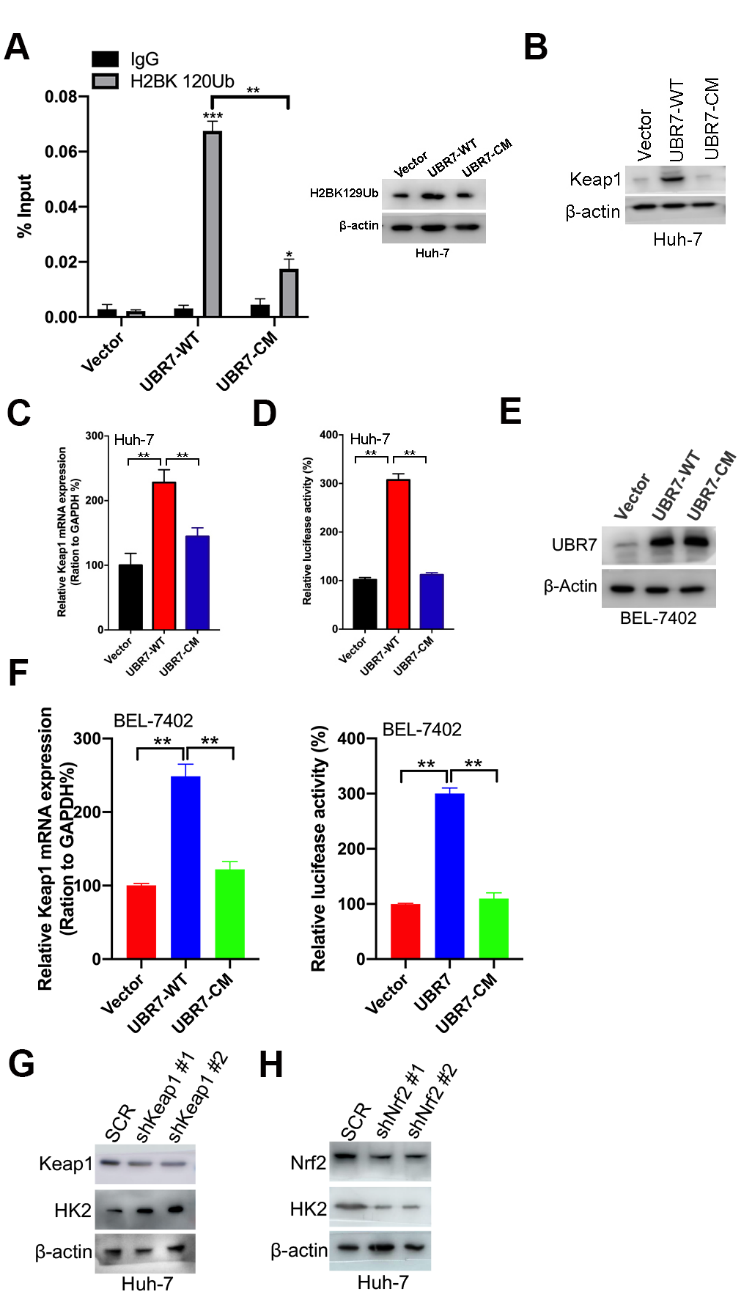
**Fig. 8** **UBR7-CM affects Keap1 expression.** (**A**) Bar graph of qPCR enrichment of H2BK120Ub ChIP in Huh-7 cells expressing UBR7-WT or UBR7-CM. (**B**, **C**) Keap1 expression levels in Huh-7 cells overexpressing UBR7-WT or UBR7-CM. (**D**) Fluorescein reporter gene analysis UBR7 binds to H2B on Keap1 in Huh-7 cells overexpressing UBR7-WT or UBR7-CM. (**E**) Western blot was used to detect the expression levels of UBR7 in BEL-7402 that overexpression UBR7-WT or UBR7-CM. (**F**) Left: BEL-7402 cells overexpressing UBR7-CM were used to detect Keap1 mRNA levels. Right: Fluorescein reporter gene analysis UBR7 binds to H2B on Keap1 in BEL-7402 cells overexpressing UBR7-CM. (**G**, **H**) Immunoblot was used to detect the protein expression levels of HK2 in Huh-7 cells knocked out Keap1 or Nrf2. Data are shown as mean ± SD of three independent experiments. **p < 0.01, ***p < 0.001.


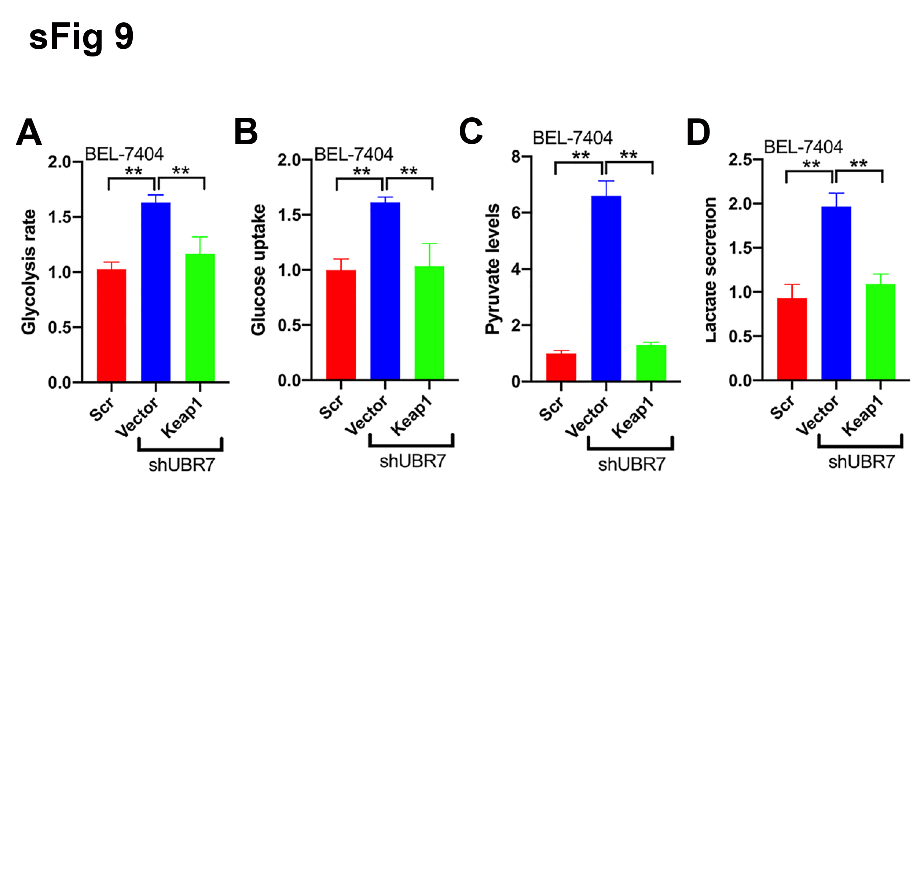


**Supplementary Fig. 9** **Keap1 affects the regulation of glycolysis by UBR7.** Glycolysis rate (**A**), glucose uptake (**B**), Pyruvate levels (**C**) and Lactate secretion levels (**D**) were detected in BEL-7404 cells overexpressing Keap1 and knocking out UBR7. Data are shown as mean ± SD of three independent experiments. **p < 0.01.


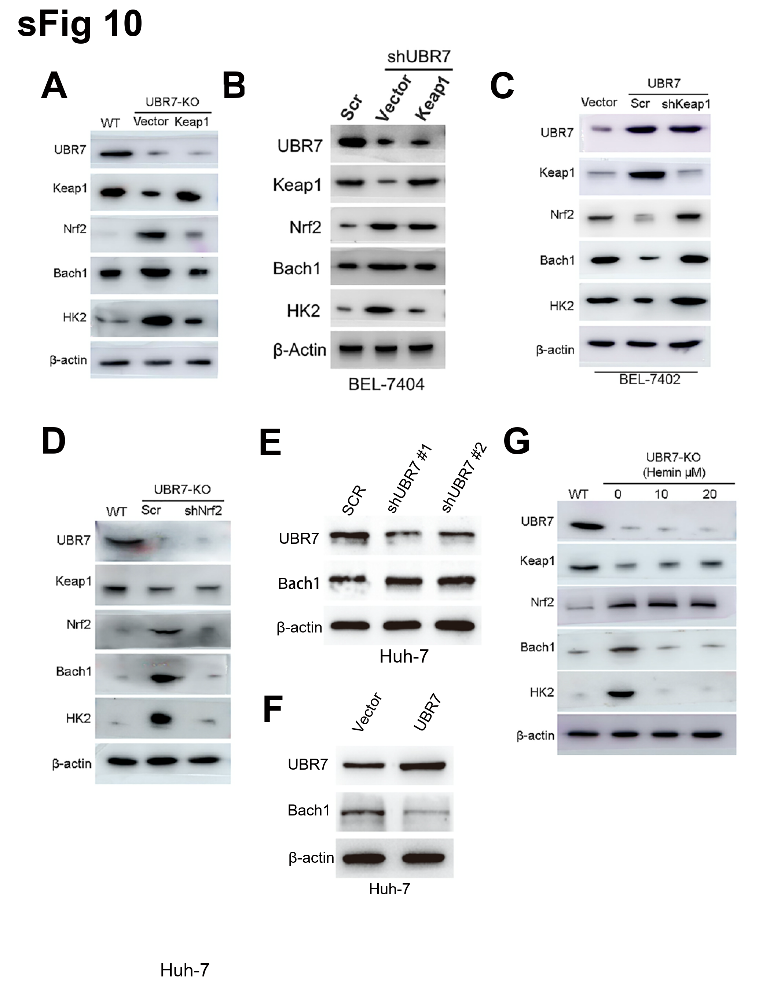


**Supplementary Fig. 10** (**A**) Immunoblot was used to detect the protein expression levels of Keap1, Nrf2, Bach1 and HK2 in UBR7-KO cells overexpressed Keap1. (**B**) Western blot was used to detect the expression levels of UBR7, Keap1, Nrf2, Bach1 and HK2 proteins in BEL-7404 cells that knocked out UBR7 and overexpressed Keap1. (**C**) The protein expression levels of Keap1, Nrf2, Bach1 and HK2 were detected by immunoblot in BEL-7402 cells overexpressing UBR7 and knocking out Keap1. (**D**) Immunoblot was used to detect the protein expression levels of Keap1, Nrf2, Bach1 and HK2 in UBR7-KO cells knocked out Nrf2. (**E**) Western blotting analysis of Bach1 expression levels upon UBR7 silencing in Huh-7 cells. (**F**) Western blotting analysis of Bach2 expression levels upon UBR7 silencing in Huh-7 cells. (**G**) Immunoblot was used to detect the protein expression levels of Keap1, Nrf2, Bach1 and HK2 in UBR7-KO cells treated with Hemin.


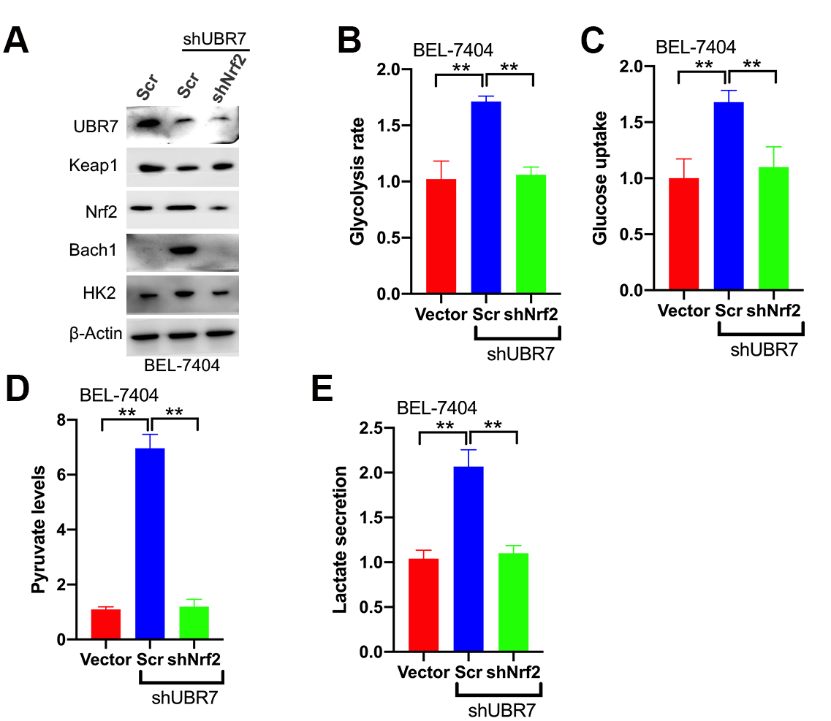


**Supplementary Fig. 11** **Nrf2 affects the regulation of glycolysis by UBR7.** (**A**) Western blot was used to detect the expression levels of UBR7, Keap1, Nrf2, Bach1 and HK2 proteins in BEL-7404 cells that knocked out UBR7 and Nrf2. Glycolysis rate (**B**), glucose uptake (**C**), Pyruvate levels (**D**) and Lactate secretion levels (**E**) were detected in knocked out UBR7 and Nrf2 BEL-7204 cells knocked out UBR7. Data are shown as mean ± SD of three independent experiments. **p < 0.01.

**Supplementary**
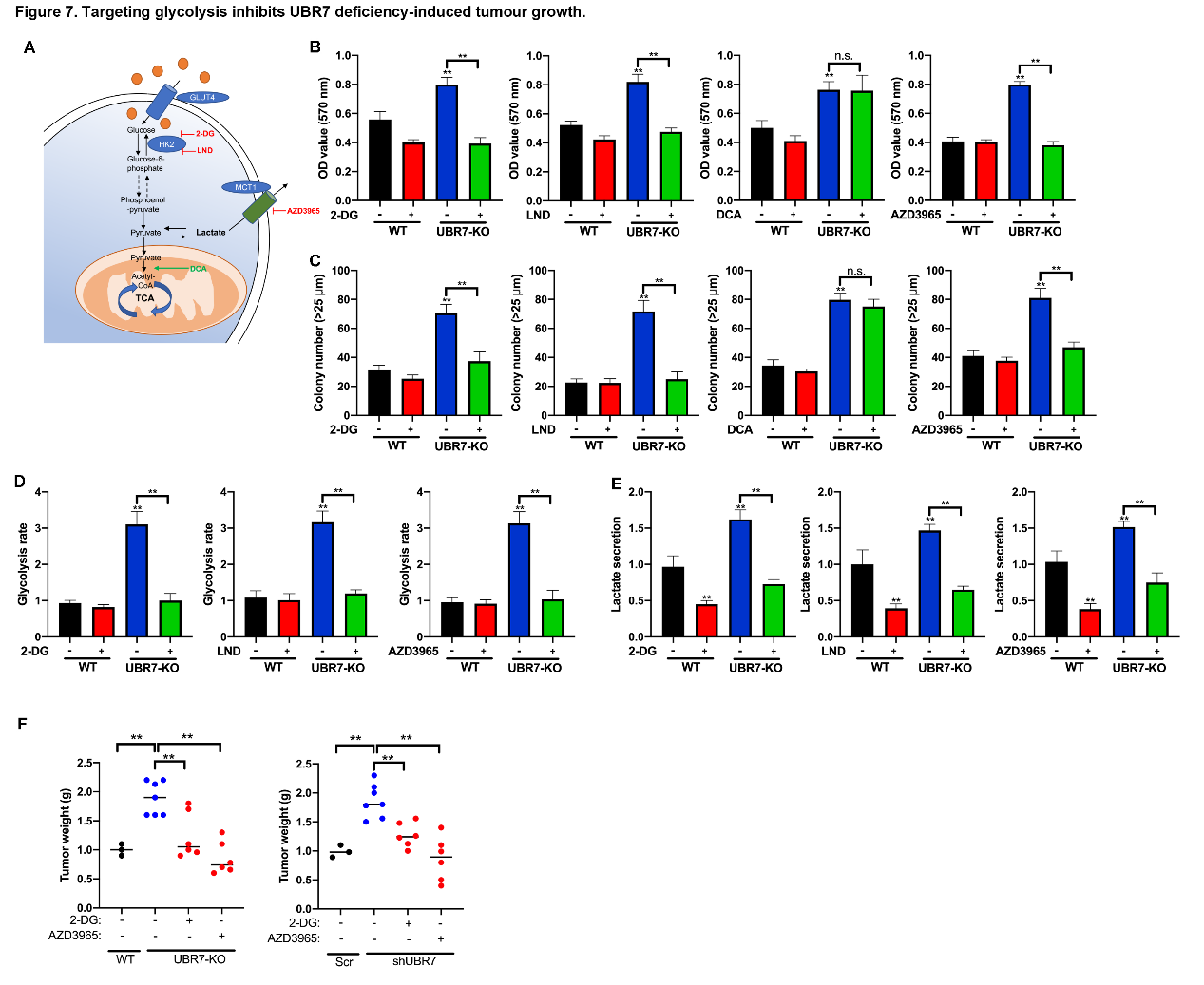
**Fig. 12 Targeting glycolysis inhibits UBR7 deficiency-induced tumour growth.** (**A**)Various enzymes and their inhibitors (red) and activator (green) in the sugar metabolism pathway. (**B**, **C**)2-DG, LND, DCA, and AZD3965 inhibitors were used to treat UBR7-KO (Alb-Cre;Ubr7^fl/fl^) cells to detect cell viability and cloning ability. (**D, E)** 2-DG, LND and AZD3965 treated UBR7-KO cells to detect glycolysis levels and lactate secretion levels. (**F)** After treatment with 2-DG and AZD3965, UBR7-KO cells and UBR7 knockout cells affected the size of xenograft tumours. Data are shown as mean ± SD of three independent experiments. **p < 0.01.

**Supplementary**
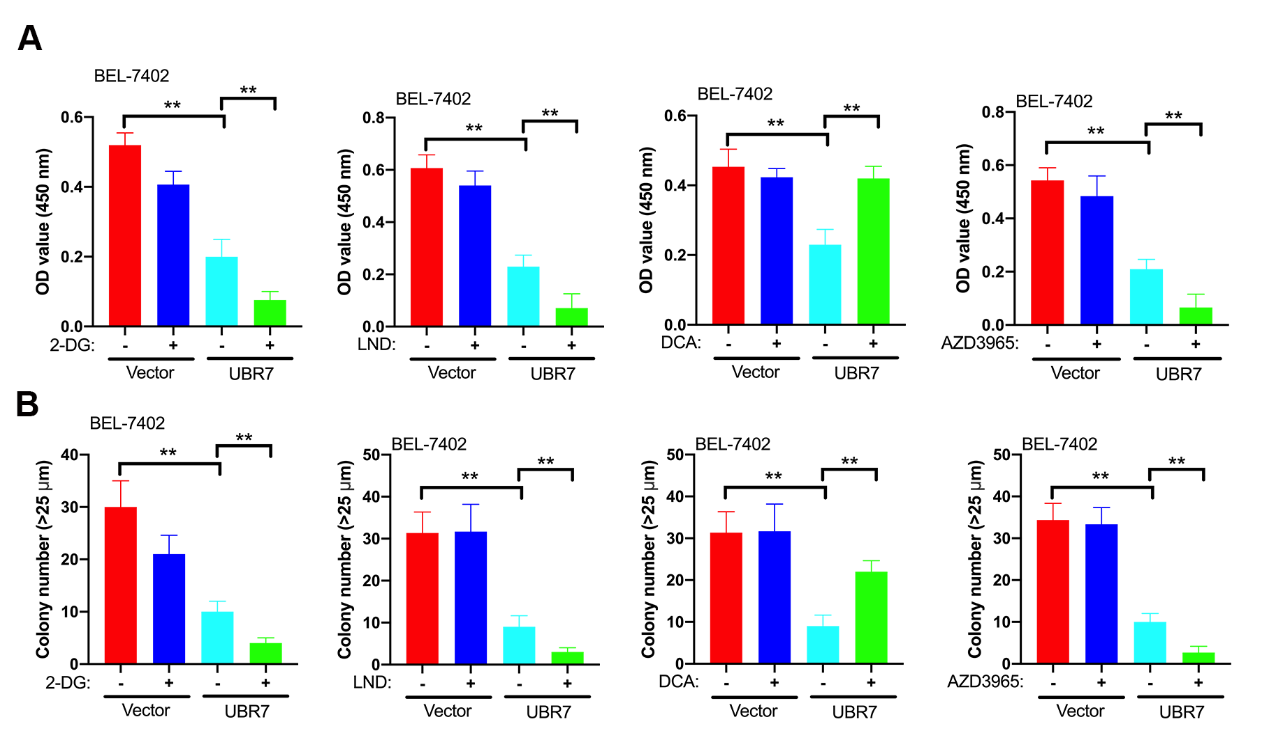
**Fig. 13** **Targeting glycolysis inhibits UBR7 overexpression-induced cell growth.** 2-DG, LND, DCA and AZD3965 treated BEL-7402 overexpressing UBR7 to detect cell viability (**A**) and clone formation (**B**). Data are shown as mean ± SD of three independent experiments. **p < 0.01.


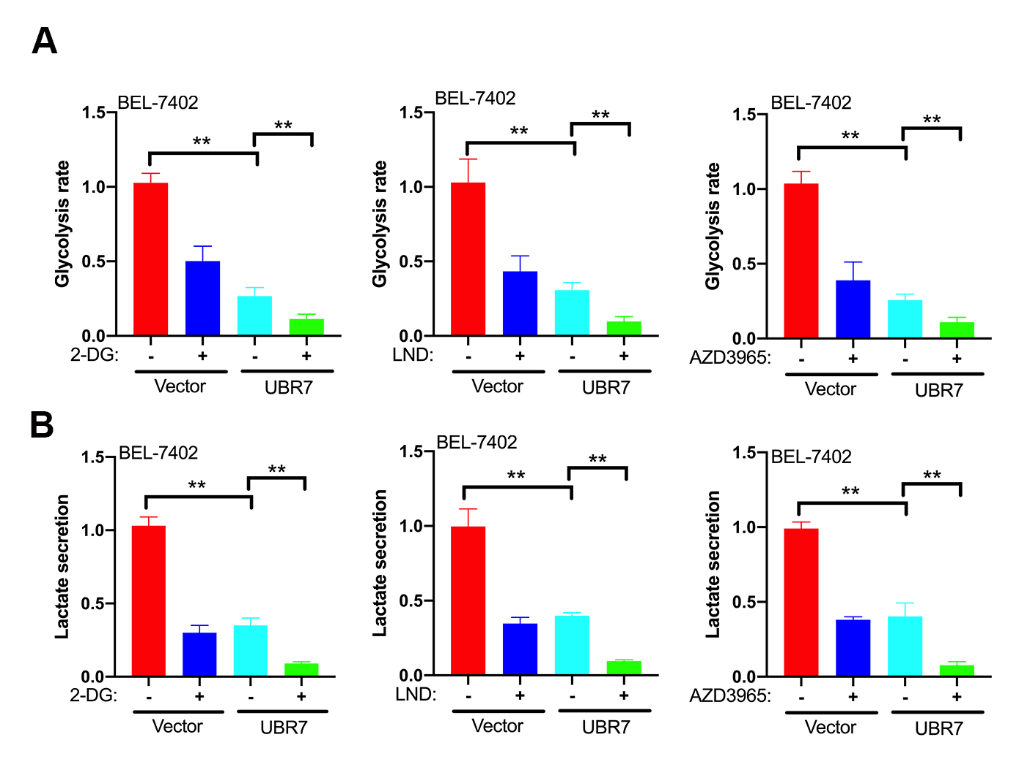


**Supplementary Fig. 14** **Targeting glycolysis inhibits UBR7 overexpression-induced glycolysis.** 2-DG, LND, DCA and AZD3965 treated BEL-7402 overexpressing UBR7 to detect glycolysis rate (**A**) and lactate secretion (**B**). Data are shown as mean ± SD of three independent experiments. **p < 0.01.

**Supplementary**
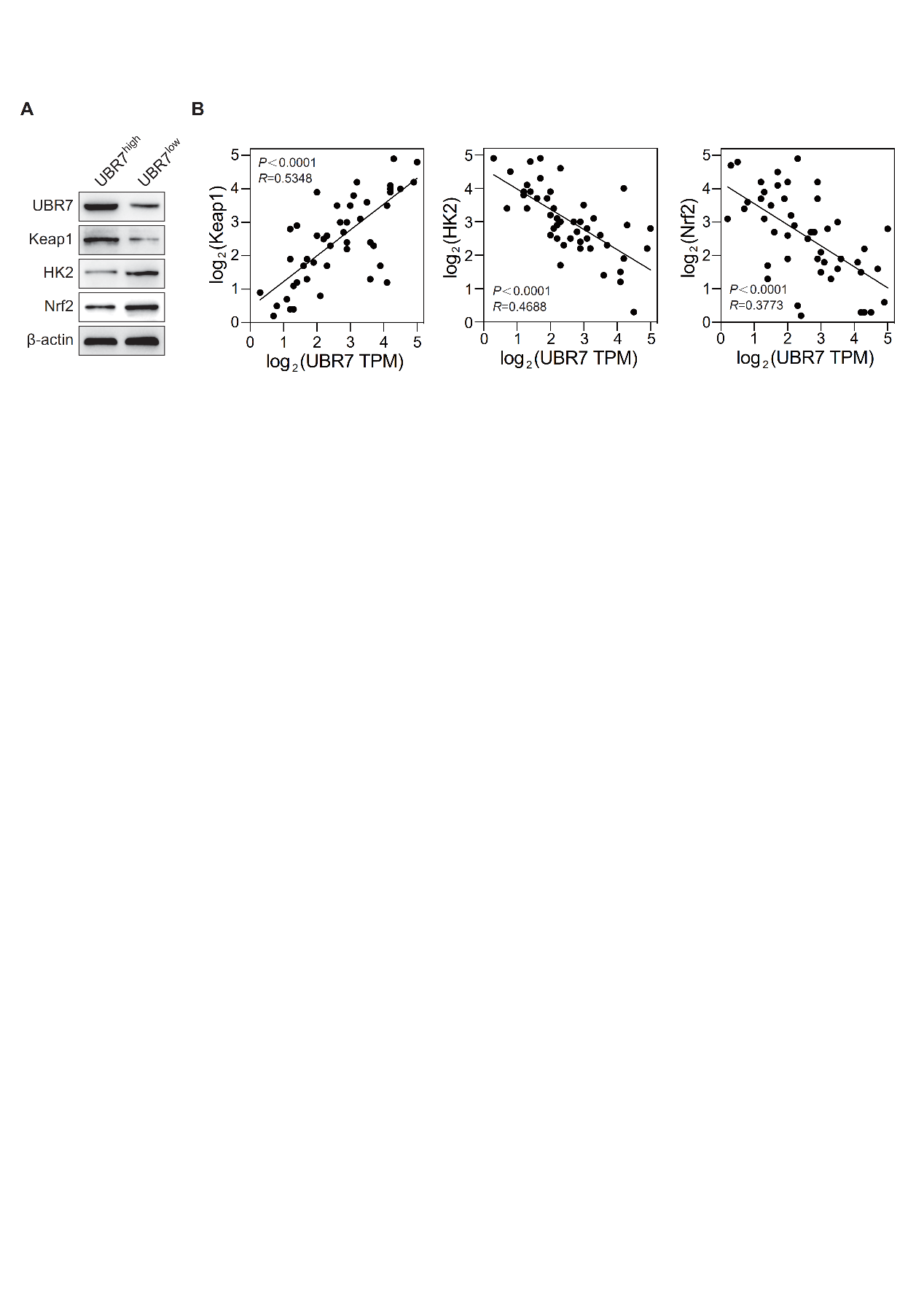
**Fig. 15** **The correlation of UBR7-Keap1/Nrf2/HK2 axis in HCC patients.** (**A**) Protein levels of UBR7, Keap1, HK2 and Nrf2 were determined by western blot from clinical liver tissue. (**B**) Correlation between UBR7 and Keap1/Nrf2/HK2 in HCC patients. The pair-wise Pearson correlation coefficient and the corresponding p-value between two genes were calculated.
